# Single-channel kinetics of BK (Slo1) channels

**DOI:** 10.3389/fphys.2014.00532

**Published:** 2015-01-21

**Authors:** Yanyan Geng, Karl L. Magleby

**Affiliations:** ^1^Department of Physiology and Biophysics, University of Miami Miller School of MedicineMiami, FL, USA; ^2^Neuroscience Program, University of Miami Miller School of MedicineMiami, FL, USA

**Keywords:** Markov models, channel gating, 50-state model, dependency

## Abstract

Single-channel kinetics has proven a powerful tool to reveal information about the gating mechanisms that control the opening and closing of ion channels. This introductory review focuses on the gating of large conductance Ca^2+^- and voltage-activated K^+^ (BK or Slo1) channels at the single-channel level. It starts with single-channel current records and progresses to presentation and analysis of single-channel data and the development of gating mechanisms in terms of discrete state Markov (DSM) models. The DSM models are formulated in terms of the tetrameric modular structure of BK channels, consisting of a central transmembrane pore-gate domain (PGD) attached to four surrounding transmembrane voltage sensing domains (VSD) and a large intracellular cytosolic domain (CTD), also referred to as the gating ring. The modular structure and data analysis shows that the Ca^2+^ and voltage dependent gating considered separately can each be approximated by 10-state two-tiered models with five closed states on the upper tier and five open states on the lower tier. The modular structure and joint Ca^2+^ and voltage dependent gating are consistent with a 50 state two-tiered model with 25 closed states on the upper tier and 25 open states on the lower tier. Adding an additional tier of brief closed (flicker states) to the 10-state or 50-state models improved the description of the gating. For fixed experimental conditions a channel would gate in only a subset of the potential number of states. The detected number of states and the correlations between adjacent interval durations are consistent with the tiered models. The examined models can account for the single-channel kinetics and the bursting behavior of gating. Ca^2+^ and voltage activate BK channels by predominantly increasing the effective opening rate of the channel with a smaller decrease in the effective closing rate. Ca^2+^ and depolarization thus activate by mainly destabilizing the closed states.

## Introduction

Large conductance Ca^2+^- and voltage activated K^+^ channels (also referred to as Slo1 or maxi K^+^ channels) are widely distributed. BK channels (for big conductance) have an unusually high conductance for such a K^+^ selective channel of ~300 pS in symmetrical 150 mM KCl. Their joint activation by depolarization and Ca^2+^ (Marty, 1981; Pallotta et al., 1981; Latorre et al., 1982) provides a negative feed-back system to drive the membrane potential more negative, which would then close both the open BK channels and also the voltage dependent Ca^2+^ channels that are often co-localized with BK channels (Robitaille et al., 1993; Wang et al., 2001). Through this negative feedback mechanism, BK channels are involved in many physiological processes (Vergara et al., 1998). Dysfunction of BK channels can lead to diseases such as autism and mental retardation (Laumonnier et al., 2006), epilepsy (Du et al., 2005), asthma (Seibold et al., 2008), cerebellar ataxia (Sausbier et al., 2004), and hypertension (Sausbier et al., 2005).

There have been a number of recent reviews on the gating mechanisms of BK channels (Magleby, 2003; Cox, 2005, 2007; Latorre and Brauchi, 2006; Cui et al., 2009; Latorre et al., 2010; Lee and Cui, 2010; Horrigan, 2012; Contreras et al., 2013; Hoshi et al., 2013). To supplement these reviews, we will take a different approach for our contribution to the Frontiers in Physiology Research Topic: BK channels: integrators of cellular signals in health and disease. Our review will focus specifically on what selected single-channel studies have revealed about the Ca^2+^- and voltage-dependent gating of BK channels. The level of presentation will be introductory. Beta subunits, Mg^2+^ and other modulators, as well as channel conductance, and selectivity are not considered. Representative figures of single-channel data and analysis are included so that the review is relatively self-contained. Those who seek further information about gating mechanism after reading this introductory single-channel review can start with the extensive reference lists found in the reviews listed above.

## The question

**Figure 1:**
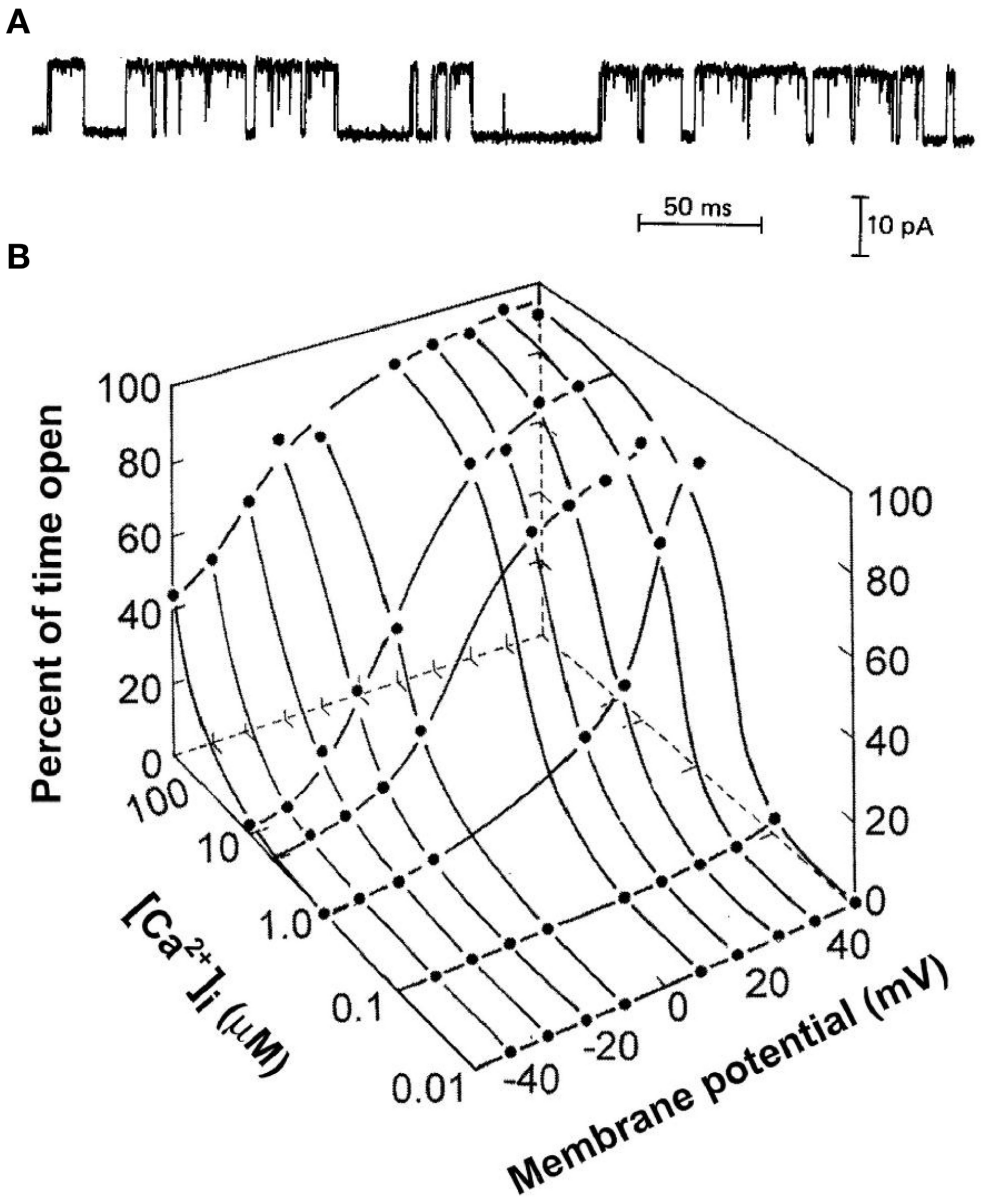
**Synergistic activation of BK channels by Ca^2+^ and depolarization. (A)** A current record from a membrane patch with a single BK channel for 7.5 μM intracellular Ca^2+^ and +30 mV (from McManus and Magleby, 1988). Channel opening is indicated by upward current steps and channel closing by downward current steps. **(B)** Plot of open probability Po as a function of Ca^2+^ and voltage (from Barrett et al., 1982). All references to Ca^2+^ in this paper are for Ca^2+^ at the intracellular membrane.

Figure 1A presents the current through a single BK channel in a small patch of membrane, recorded with the patch clamp technique (Hamill et al., 1981). In this case, the inside of the excised patch of membrane in the tip of a glass pipette is exposed to the bath solution, allowing the solution at the inner membrane surface to be easily changed. The upward and downward steps in the current reflect channel opening and channel closing, respectively. Measuring the current record at half open amplitude gives a record of successive open and closed interval durations that allow the calculation of open probability, Po, from the total open time divided by the sum of the total open time plus the total closed time. The successive interval durations also give information about the underlying gating mechanism of the channel. The gating is complex with intervals over wide ranges of durations. Figure 1B shows that BK channels are activated synergistically by both Ca^2+^ and depolarization. In resting skeletal muscle with voltage of −80 mV and intracellular Ca^2+^ < 0.1 μM, BK channels seldom open. Increasing both Ca^2+^ and depolarization are then required to activate the channels under physiological conditions. This review considerers the question of what types of kinetic gating mechanisms can account for the single-channel current records for the activation of BK channels by Ca^2+^ and voltage.

## Modular structure of BK channels

**Figure 2:**
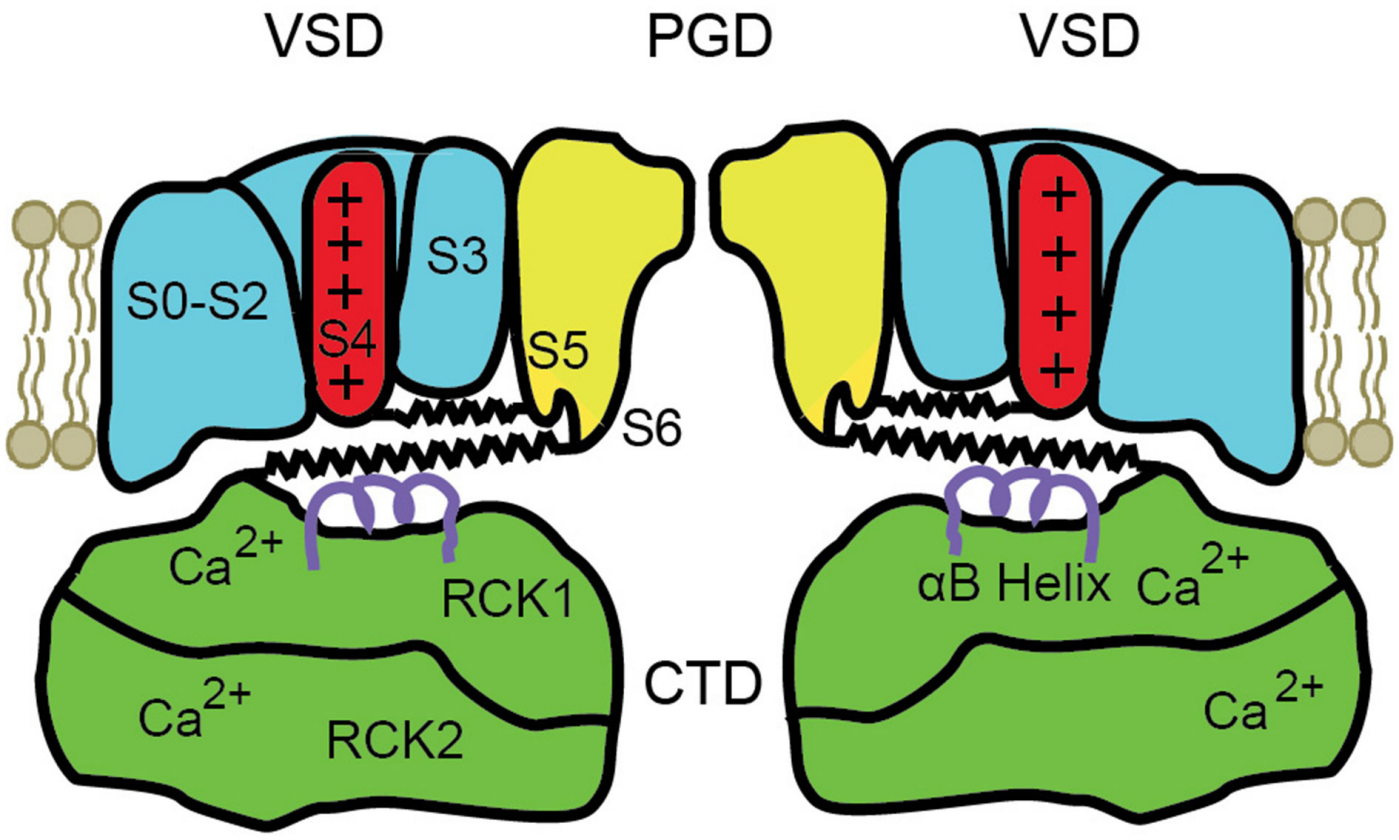
**Schematic of the modular structure of a BK channel**. The central pore-gate domain (PGD) formed from S5 to S6 of each of the four subunits forms the gates and conducting pore of the channel. Two of the four voltage sensor domains (VSD) that surround the central PGD are shown. The cytosolic domain (CTD) forms a large intracellular gating ring comprised of four RCK1 domains and four RCK2 domains, of which only two of each are shown. Each RCK1 domain contains a high affinity RCK1 Ca^2+^ binding site and each RCK2 domain contains a high affinity Ca^2+^ bowl binding site. Four S4–S5 linkers connect S4 in the VSDs to the PGD (upper springs) and four RCK1-S6 linkers connect the gating ring to the PGD (lower springs), shown for two subunits. Ca^2+^ binding expands the gating ring to pull on the RCK1-S6 linkers and can also elevate the αB helices (purple helices) under the VSDs (Yuan et al., 2012). See text for references and detailed explanations.

A schematic diagram of the modular structure of a BK channel is shown in Figure 2. Only two of the four subunits are shown with front and back subunits removed. BK channels are comprised of four transmembrane voltage sensor domains (VSD) surrounding a central pore gate domain (PGD), and a large cytosolic domain (CTD), as shown schematically in Figure 2. The CTD is also referred to as the gating ring (Jiang et al., 2002). Each VSD is formed from S0 to S4 transmembrane segments of a single subunit. The central PGD is formed from S5 and S6 from each of the four subunits, with the pore loops between S5 and S6 forming the selectivity filter that excludes anions, Na^2+^, Ca^2+^, and Mg^2+^, allowing K^+^ to pass through the channel at a high rate. The gating ring is assembled from the RCK1 and RCK2 domains (regulators of the conductance of K^+^) of each of the four subunits (Yuan et al., 2010), forming a ring with a central opening. The gating ring is attached to the PGD by four RCK1-S6 linkers, indicated for two subunits by the lower spring on each side of the drawing. The RCK1 domain of each subunit contains a high affinity Ca^2+^ binding site termed the RCK1 site (K_0.5_ 4–17 μM), and the RCK2 domain contains a second high affinity Ca^2+^ binding site called the Ca^2+^ bowl (K_0.5_ 2–4 μM) (Schreiber and Salkoff, 1997; Bao et al., 2002; Shi et al., 2002; Xia et al., 2002; Zeng et al., 2005; Zhang et al., 2010). Removing the gating ring by severing the RCK1-S6 linkers at the RCK1 end removes all Ca^2+^ and Mg^2+^ sensitivity, converting the BK channel into a purely voltage sensitive channel (Budelli et al., 2013). Ca^2+^ binding to the gating ring effectively expands the gating ring, pulling on the RCK1-S6 linkers to activate the channel (Pico, 2003; Niu et al., 2004; Yuan et al., 2010, 2012). The above information indicates that the gating ring is the module of the channel responsible for Ca^2+^ sensitivity.

Depolarization of BK channels elevates the S4 transmembrane segment in the VSD, pulling on the S4–S5 linker, which acts on the PGD to open the channel (Ma et al., 2006; Hoshi et al., 2013; Zhang et al., 2014). S4–S5 linkers for two subunits are shown as the upper springs in Figure 2. Charges in S1–S3 also contribute to voltage sensitivity (Ma et al., 2006). The convergence of the action of depolarization and Ca^2+^ on the PGD through the RCK1-S6 linkers and the S4–S5 linkers provides an explanation for the synergistic activation of BK channels by both depolarization and Ca^2+^. Mg^2+^ modulates the activity of BK channels through four low affinity sites (K_0.5_ 2–4 mM), each located between the cytosolic side of a VSD and the top of an RCK1 domain in the gating ring (Hu et al., 2003; Yang et al., 2007). This review will focus on Ca^2+^ and voltage activation.

Where is the gate? The term gating is used to refer to all the processes involved in channel activity, including Ca^2+^ and voltage induced conformational changes as well as the actual opening and closing of the gate(s) in the conduction pathway. The physical location and mechanism by which the gate blocks the pore in BK channels is not clear, but the gate may be located deep in the inner vestibule of the PGD just below the selectivity filter, such that closing arises from movement (possible rotation) of the four S6 transmembrane segments to form a hydrophobic region that physically obstructs the deep pore to close or almost close the channel (Wilkens and Aldrich, 2006; Chen et al., 2014). As a possible second step, the hydrophobic region may then exclude water, leading to the formation of a water free region (a bubble) that completely blocks the movement of ions (Roth et al., 2008). Consistent with this two-step possibility, Ferguson et al. (1993) found that transitions from fully open to fully closed (and also transitions from fully closed to fully open) often proceeded through a very brief duration almost closed subconductance state with a conductance about 5% of the fully open state. The channel could also reopen from the brief duration subconductance states giving rise to very brief flicker (almost) closed states.

## Discrete state Markov models as gating mechanisms

Discrete state Markov (DSM) models, after Russian mathematician Andrey Markov, have proven highly useful to describe single-channel gating (Colquhoun and Hawkes, 1982, 1995b; Magleby and Pallotta, 1983b; McManus and Magleby, 1991; Gil et al., 2001; Lape et al., 2008). These models assume that channels gate by moving among different conformational and/or agonist bound states of the channel protein. Open and closed states of the channel represent two different conformational states. A channel with all four voltage sensors deactivated (down) is in a different conformational state than a channel with three voltage sensors deactivated and one activated (up). A channel with no bound Ca^2+^ is in a different bound state than a channel with one bound Ca^2+^, which is in a different state than a channel with two bound Ca^2+^.

**Figure 3:**
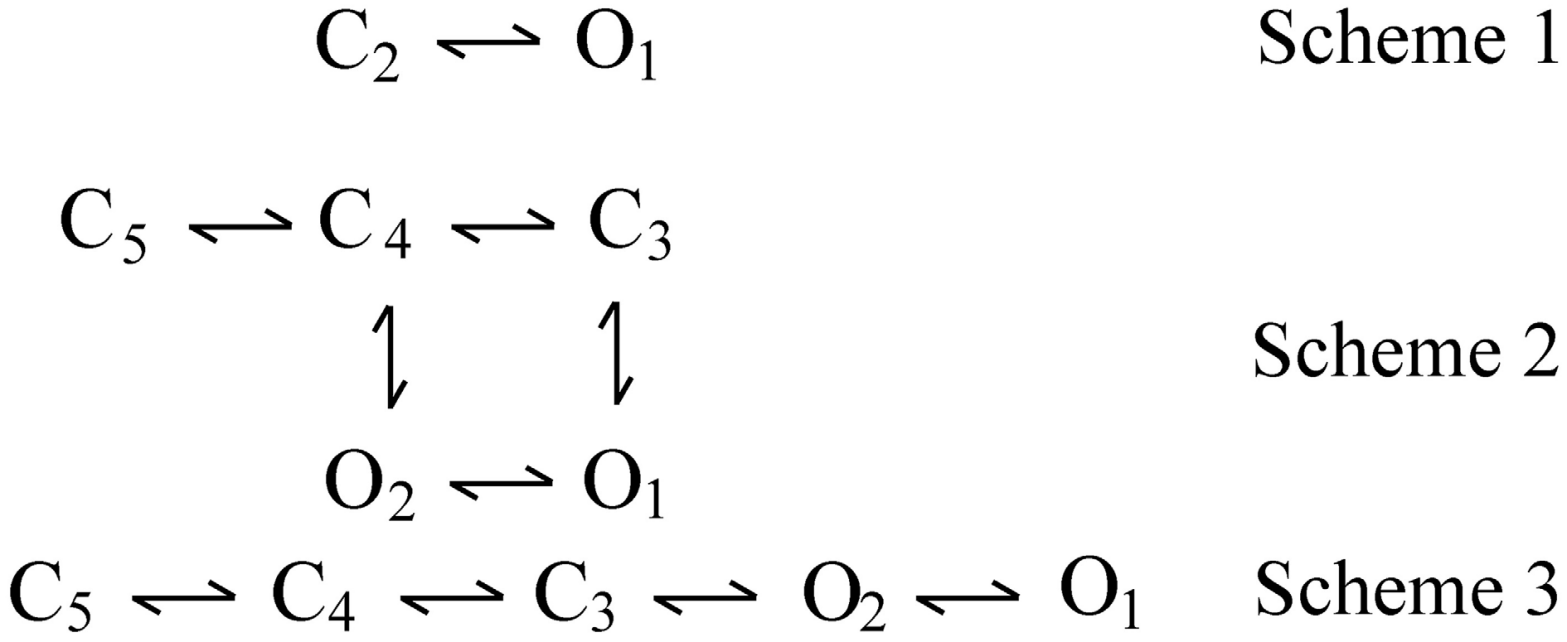
**Examples of discrete state Markov (DSM) models with limited numbers of states**. C and O are closed and open states, respectively. Scheme 1 is the simplest model that gates. Scheme 2 with two gateway states (C_3_ and C_4_) connecting open and closed states would typically generate correlations between adjacent open and closed interval durations. Scheme 3 with one gateway state (C_3_) would not generate such correlations (Fredkin et al., 1985; Magleby and Song, 1992; Colquhoun and Hawkes, 1995b).

Three examples of DSM models are shown by kinetic Schemes 1–3 in Figure 3, where open (O) and closed (C) states are indicated and each state is identified by a state number. Scheme 1 is the simplest DSM model that gates. Whereas most WT channels gate in large numbers of states, Cukierman et al. (1997) constructed a channel from two gramicidin A molecules covalently linked with a dioxolane ring that gates as the two-state model in Scheme 1. Schemes 2 and 3 each have two open and three closed states, but are different gating mechanisms because the connections (transition pathways) between the states are different.

The assumptions for DSM models as applied to gating of BK channels are: (1) the time required for the transition between two connected states is insignificant compared to the time spent in either state, (2) the rate constants for the transitions between states remain constant (in time) for constant experimental conditions, and (3) voltage and Ca^2+^ gate the channel by changing the effective rates for the transitions between states. It follows from (2) that the rate constants for leaving a state are independent of the previous sequence of events before entering the state, and are also independent of the duration of time already spent in the state. For this reason Markov models are often referred to as memory less processes. However, it is not the model but the rate constants that are memory less. Markov models can have memories defined by the kinetic schemes and rate constants, leading to correlations in the data, as will be explained later.

## Dwell-times in states are exponentially distributed in DSM models

For DSM models (and also for the decay of radioisotopes), the dwell-times (lifetimes) in a state are a random (stochastic) variable, described by an exponential distribution, where the probability (density), *f*(*t*), of observing a dwell-time with duration *t* decays exponentially with *t*, such that

(1) f(t)=(1/τ)exp(−t/τ)

where *t* is the duration of a dwell-time in the state, τ is the time constant of the distribution, given by the time for the distribution to decline to 1/e (0.368) of its initial magnitude, and 1/τ is the magnitude of the distribution at time 0. τ is also given by the mean of all the intervals in the exponential distribution. The product of τ times the magnitude of the exponential at time 0 gives the area of the exponential. The probability (density) function given by Equation (1) is normalized to an area of 1.0 by setting the magnitude to 1/τ. Increasing τ 10-fold then reduces the magnitude 10-fold to retain an area of 1.0.

Why are dwell-times in a Markov state exponentially distributed? Converting a rate constant to a probability indicates a small probability of leaving a state in each small increment of time. For example, a rate constant of 1/s away from a state converts to a probability for leaving the state of 10^−3^/ms, 10^−6^/μs, or 10^−9^/ns. A small but constant probability of leaving a state per unit time leads to an exponential distribution of the times spent in the state (Colquhoun and Hawkes, 1995b). As a thought experiment, imagine that the channel possesses a true die with 10^9^ sides which it throws once every nanosecond. If side 1 out of the 10^9^ sides comes up, then the channel makes a conformational change. If any of the other 999,999,999 sides come up, then the channel throws the die again without memory of previous throws, and continues to do so until side 1 comes up, at which point it makes a conformational change. Mechanistically, during each brief moment of time (10^−9^ s), the thermal fluctuations in the channel have only a low probability of providing sufficient energy to move the present conformation over a thermally fluctuating energy barrier into the next state, or alternatively, during each brief moment of time the channel very rapidly samples many different conformations due to thermal fluctuations, with only a very low probability of reaching a different stable conformation state. See Frauenfelder (2014) for discussion of protein motions.

The engineered two-state channel of Cukierman et al. (1997) discussed in the previous section was identified as gating in two states from the single exponential distributions for open and closed intervals. The distributions of open times for sodium channels are described by a single exponential, suggesting a single open state (Horn and Vandenberg, 1984). For channels with multiple open and closed states, dwell-times in any given state are still exponentially distributed for DSM models, but the separate dwell-time distributions of all open and all closed interval durations are now comprised of the sums of exponentials, as will be described in later sections.

## Calculation of state lifetimes and probabilities for different transitions

Rate constants for DSM models are identified by state numbers, such that *k*_i–j_ is the rate constant for the transition from state *i* to state *j*. The mean lifetime (dwell-time) in any state *i* can be calculated from the rate constants (Colquhoun and Hawkes, 1995b) using

(2) $$\begin{array}{l} \text{Mean lifetime of state}\\ \;i=1/\left(\sum \text{ rate constants away from state }i\right) \end{array}$$

where (Σ rate constants away from state *i*) gives the effective rate constant for leaving state *i*. For example, the mean lifetime of C_2_ in Scheme 1 is given by 1/*k*_2−1_, the mean lifetime of C_5_ in Scheme 2 is given by 1/*k*_5−4_, and the mean lifetime of C_4_ in Scheme 2 is given by 1/(*k*_4−2_ + *k*_4−3_ + k_4−5_). It follows from rearranging Equation (2) that the rate constants can be determined directly for the two-state model given by Scheme 1 for data in which all open and closed intervals are detected from the inverse of the mean lifetimes of the open and closed intervals

(3) $$k_{1-2}=1/{\text{O}}_{1}$$

(4) $$k_{2-1}=1/{\text{C}}_{2}$$

For Scheme 1, with only one transition pathway between the states, which state is entered next is a given. For more complex models like Schemes 2 and 3, the probability of making a transition from state *i* to state *j*, *P*_i−j_ is

(5) $$P_{\text{i}-\text{j}}=k_{\text{i}-\text{j}}/\left(\sum \text{ rate constants away from state}\;i\right)$$

For Scheme 2, *P*_5−4_equals *k*_5−4_/*k*_5−4_ equals 1, and *P*_4−2_ equals *k*_4−2_/(*k*_4−2_ + *k*_4−3_ + *k*_4−5_). For Scheme 3 *P*_2−1_ equals *k*_2−1_/(*k*_2−1_ + *k*_2−3_).

The connected states C_5_-C_4_-C_3_ in Schemes 2 and 3 form compound closed states, with each of the states in the compound state having the same closed current level, so that the state transitions within the compound closed states are hidden from direct experimental observations. Examples of hidden transitions within the compound closed state in Scheme 2 would be: O_1_-C_3_-C_4_-O_2_ and O_2_-C_4_-C_3_-C_4_-C_5_-C_4_-O_2_. The same would apply to transitions among the compound open states, O_1_-O_2_, which in BK channels would have the same open current level. Consequently, compound openings such as C_4_-O_2_-O_1_-O_2_-C_4_ for Scheme 2 and C_3_-O_2_-O_1_-O_2_-C_3_ for Scheme 3, would all have the same open current levels with the open–open transitions hidden. In spite of the hidden transitions, information about the numbers of closed and open states, the transition pathways among the states, the probabilities of the various transitions pathways, and the mean lifetimes of the states are reflected in the successive open and closed interval durations, which reveal the closed and open dwell-time distributions and also the 2D dwell-time distributions and the correlations between open and closed interval durations (see graphical demonstrations in (Magleby and Song, 1992; Colquhoun and Hawkes, 1995b; Rothberg et al., 1997; Rothberg and Magleby, 1998a). Because state transitions are hidden within compound states, DSM models are also referred to as hidden Markov models.

## Describing dwell-time distributions by the sums of exponentials

Even though transitions among states in a compound state are hidden, each of these states will lead to the generation of an exponential component in the distribution of interval durations (Colquhoun and Hawkes, 1982, 1995b). For *n_c_* closed and *m_o_* open states in a DSM model, the dwell-time distribution of all closed intervals would then consist of the sum of *n_c_* exponential components, and the dwell-time distribution of all open intervals would consist of the sum of *m_o_* open exponentials components, although all exponentials may not be detected because of closely spaced or identical time constants, or areas too small to detect. Distributions described by sums of exponentials are often referred to as being described by mixtures of exponentials because it is not possible to assign any single interval in the summed distribution to any given exponential component, except in terms of probabilities. A first step often used in the analysis of single-channel data is to fit open and closed dwell-time distributions with sums of exponentials to determine the numbers of significant exponential components, which then provides an estimate of the minimum number of open and closed states required in a DSM, such that,

(6) $$f(t)=\sum_{k=1}^{N}\left(a_{k}/\tau_{k})\exp(-t/\tau_{k}\right)$$

where *f*(*t*) is the dwell-time distribution arising from the sums of exponential components, *N* is the number of summed exponentials, *a_k_* is the area (the fraction of the total area of the dwell-time distribution) of exponential *k*, *τ_k_* is the time constant of exponential *k*, and the quotient *a*_k_/τ_k_ gives the magnitude of exponential *k* at 0 time (Landowne et al., 2013).

A common misconception in the interpretation of dwell-time distributions is to assume a one-for-one relationship between exponential components and states, such that, the dwell-times in a specified state give rise to the dwell-times in a single exponential component. Such a relationship is seldom the case when compound states are involved, as each state in a compound state can make contributions to all of the exponential components arising from the compound state. The relationship between components and states is fascinating, being both paradoxical or predictable depending on the ratios of the various rate constants connecting the states (Shelley and Magleby, 2008). Fortunately, the methods used to determine rate constants and rank models circumvent the need to know the complexities of these relationships. Nevertheless, to discuss exponential components in terms of states, which is often done, requires understanding the relationship between components and compound states.

## Estimating parameters and ranking kinetic gating mechanisms

**Figure 4:**
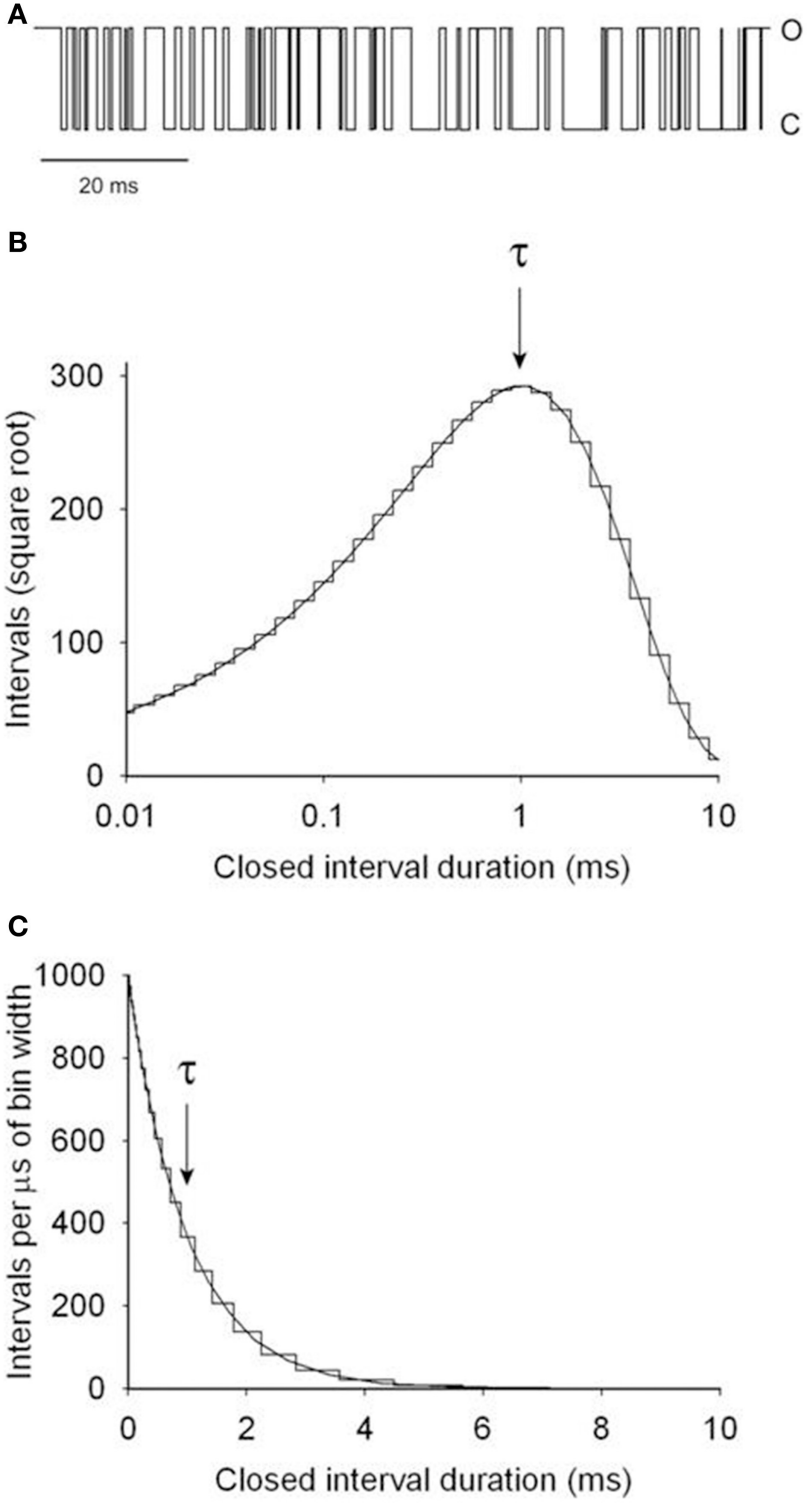
**Simulated single channel kinetics for the two-state Scheme 1**. **(A)** A simulated single-channel current record for Scheme 1 with opening and closing rate constants of 1000/s. **(B)** Dwell-time distribution of the closed interval durations plotted with the Sigworth and Sine transformation (Sigworth and Sine, 1987). The abscissa has log binning with 10 bins per log unit. The distribution peaks at the mean interval duration of 1 ms, indicated by τ. The rate constant for opening is given by the inverse of τ. **(C)** Dwell-time distribution of the same data in part **B** on a linear plot. The intervals were corrected for the increasing bin width that occurs with log binning. The mean interval duration τ is given by the time to decay to 1/e (1/2.718 = 0.368) of the initial amplitude. The same distributions would be obtained for the open intervals. 10^6^ intervals were simulated and analyzed (from Shelley et al., 2010).

This section illustrates by example how kinetic parameters can be estimated for a two-state model and then describes the process for more complex models. Figure 4A presents a segment of a simulated single-channel record for Scheme 1 with rate constants for opening, *k*_C2−O1_, and for closing, *k*_O1−C2_, of 1000/s. The variation in successive dwell-times and the apparent drift in activity over time reflects stochastic variation in dwell-times. Measuring the durations of 10^6^ simulated closed intervals and plotting them as a frequency histogram gives the linear plot of the dwell-time distributions in part **C**, which is well-described by the exponential function (continuous line)

(7) f(t)=1000 exp(−t/τ)

where *f*(*t*) is the number of intervals per μs of bin width, *t* is interval duration, and τ is 1 ms, given by the time required for the exponential to fall to 1/e of its initial magnitude. Figure 4B is a Sigworth and Sine (1987) plot of the same data in Figure 4C. In this transform, the distribution peaks at the time constant of the exponential, providing a quick visual indication of the mean interval duration in the distribution. Log binning is used in both parts **B** and **C**, with the duration of the bins increasing logarithmically with dwell-time. With log binning the bin width remains constant on a logarithmic abscissa. The Sigworth and Sine transform plots the square root of the number of intervals per bin vs. the log of the mean duration of the intervals in each bin, and the linear plot presents the number of intervals per μs of bin width (to correct for the log binning) vs. the mean duration of intervals per bin. Log binning allows binning and plotting of dwell-times with durations ranging from microseconds to hours using less than 100 bins with constant time resolution (McManus et al., 1987). Sigworth and Sine plots, because of the uncorrected increasing bin width with log binning, can give the mistaken impression that the frequency of intervals in a dwell-time distribution first increase and then decrease, but this is not the case, as can be seen by comparison to the liner plot in Figure 4C which has been corrected for the log binning.

The 1 ms time constant of decay in Figures 4B,C indicates a rate constant for channel opening, *k*_2–1_, of 1000/s (Equation 4). The analysis would be the same for the open intervals, giving a rate constant for channel closing, *k*_1–2_, of 1000/s. Hence, for a two state model and ideal data with unlimited time resolution, the rate constants can be determined directly from the inverse of either the time constants or the mean interval durations.

As a general experimental approach, the rate constants for Scheme 1 and for more complicated schemes would be obtained by maximum likelihood fitting of the single-channel data using Q-matrix or other methods (Colquhoun and Hawkes, 1982, 1995a,b; McManus and Magleby, 1991; Rothberg and Magleby, 1998b; Colquhoun et al., 2003; Qin, 2014). In one approach, single-channel data, in the form of 2D dwell-time distributions obtained over a range of Ca^2+^ and voltage, but with fixed conditions for each distribution, are simultaneously fitted (global fitting) with the gating mechanisms of interest (Rothberg and Magleby, 1998a). Such fitting determines the most likely rate constants and their voltage and Ca^2+^ dependence for each tested gating mechanism. Ranking the examined gating mechanisms in terms of the likelihood that the observed single-channel data were generated by the gating mechanisms, then identifies the most likely model. When ranking models, a penalty is applied for increases in the number of free parameters in the models (Rothberg and Magleby, 1999). Such global fitting of data over a range of Ca^2+^ and voltage as well as taking into account the correlation information (see later section) is essential to define models and rate constants. Without global fitting the rate constants and ranking of models can be poorly defined. The highest ranking model is not the “correct” model, which is not known, but is the most likely of all the examined models.

It is possible to determine if there is a model with the same number of states that ranks higher than the highest ranking model. The data are fitted with a generic model (Kienker, 1989) that includes all possible models for the specified number of states. Global fitting is not possible with the generic model, so each experimental condition is fitted separately with the generic model and then the log likelihoods for each experimental condition are added. If the likelihood of the highest ranked model is equal to the likelihood of the generic model for the same data, then no other model with the same number of states will be found that has likelihood higher than the highest ranked model (Rothberg and Magleby, 1998b, 1999). A comparison of the log likelihoods of the generic models to the examined models also quantifies how well the examined models account for the data (Rothberg and Magleby, 1999).

For experimental single-channel records, very brief intervals can be sufficiently attenuated by the low pass filtering used to reduce high frequency noise in the current record that the brief intervals are not detected. These missed intervals distort the single-channel data. For example, if a very brief closed interval between two longer open intervals is missed, then the missed closed interval plus the two adjacent open intervals would be detected as a single longer open interval. Alternatively, if a very brief open interval is missed, then the missed open interval plus the two adjacent closed intervals would appear as a single longer closed interval in the experimental record. Mathematical corrections for these missed events are applied when fitting experimental data to correct for the effects of the limited time resolution (Blatz and Magleby, 1986; Crouzy and Sigworth, 1990; Hawkes et al., 1992; Qin et al., 1996).

The DSM models developed in the previous sections would indicate the numbers of states, the transition pathways among the states, the rate constants for the transitions, and the voltage and Ca^2+^ dependence of the rate constants. Given such models, it is possible to calculate the expected dwell-time distributions and the macro currents over wide ranges of voltage and Ca^2+^ using Q-matrix methods (Colquhoun and Hawkes, 1982, 1995a). Macro currents are currents recorded from hundreds to thousands of channels in a macro patch of membrane or from the membrane of an intact cell. Single-channel current records can also be calculated from the DSM models through simulation using the rate constants and a random number generator to make the stochastic decisions as to specific dwell-times and state transitions. Whereas each simulated run would give a unique example of single-channel gating, combining many such runs would give a second method to calculate average single-channel and macro current kinetics and steady-state responses.

DSM models are simplifications of actual protein function. Each state in a DSM model represents large numbers of conformational substates in very rapid equilibrium due to thermally induced protein motions (McManus and Magleby, 1989; Frauenfelder, 2014). In the DSM models used here the transitions between any two connected states and the binding and unbinding of agonist (Ca^2+^) are represented with single effective rate constants, rather than with the short lifetime multi-state processes that underlie these transitions. These multistep processes are too rapid to be separated in the single-channel data. Whereas the DSM models used for gating among states do not include the multi-step processes of state transitions directly, they do give information about the dwell-times before the transitions and the different probabilities for exiting via the different transitions pathways. This information defines the DSM models and also delimits the information that will have to be accounted for by transition state theory.

In spite of the simplifications mentioned above, critical tests of Markov gating for BK and NMDA receptor channels have found that the single-channel kinetics are consistent with DSM models. The time constants of the exponentials describing the open and closed dwell-time distributions were independent of adjacent interval durations for fixed experimental conditions, as required for DSM models (McManus and Magleby, 1989; Gibb and Colquhoun, 1992). These observations of time constants independent of adjacent interval durations also indicate that the time constants of the exponentials can be accurately determined from the experimental data. Further support for Markov gating are the observations that dwell-time distributions are well-described by either single exponentials or sums of exponentials, as expected for DSM models. Fractal models can be ruled out for four examined channels, BK channels, fast Cl-channels, nicotinic AChR receptor channels, and GABA_A_ channels, as DSM models ranked significantly higher than fractal models for these four different ion channels (McManus et al., 1988).

DSM models will be referred to as gating mechanisms or schemes or simply models in the remainder of this review. Kinetic is often added to emphasize that these models can describe the dynamics of the gating over time because they contain rate constants for both forward and backward transitions. This is in contrast to equilibrium models that can only describe the steady state or equilibrium Po, and thus give no information about the time course of the gating.

## Gating mechanisms consistent with the modular structure of BK channels

Gating mechanisms are best formulated in terms of what is known about the structure of an ion channel. The modular structure in Figure 2 indicates that BK channels have: (1) four VSD modules, each with an S4 transmembrane segment that is assumed to be either deactivated (down) or activated (up); (2) a gating ring comprised of four pairs of intertwined RCK domains, each with two high affinity Ca^2+^ binding sites; and (3) a PGD that is gated by the four VSDs through the S4–S5 linkers and also by the four RCK1-S6 linkers from the gating ring. For simplicity, as is typically done for BK channels, the two high affinity Ca^2+^ binding sites per pair of intertwined RCK domains will be treated as a single site, giving four effective high affinity Ca^2+^ binding sites. This simplification is possible (but not necessarily justified) for BK channels because the Hill coefficients for plots of Po vs. Ca^2+^ typically approach, but seldom, exceed 4, as will be shown later.

**Figure 5:**
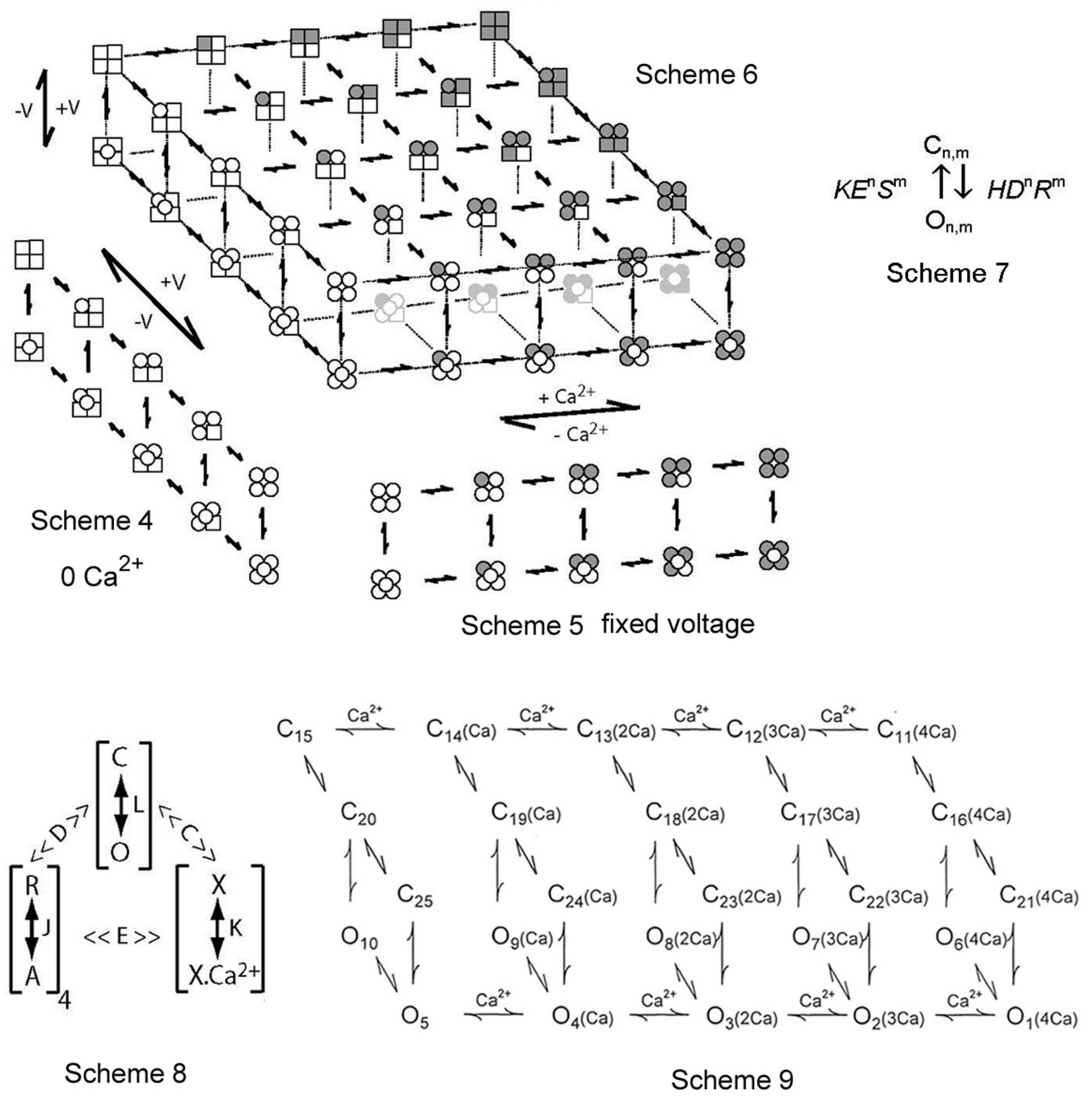
**Two-tiered gating mechanisms for BK channels**. Scheme 4 is for voltage only gating, Scheme 5 is for Ca^2+^ only gating, and Scheme 6 combines the two for joint gating by both voltage and Ca^2+^. The upper tiers are closed states and the lower tiers open states. The four subunits for each state are depicted. A filled subunit has a bound Ca^2+^ and a round subunit has an activated voltage sensor (from Rothberg and Magleby, 1999, 2000). Scheme 7 presents a constrained approach of representing the allosteric action of voltage and Ca^2+^ on the opening and closing transitions between the closed and open tiers in Schemes 4–6. Scheme 8 is a shorthand method devised by Horrigan and Aldrich (2002) to describe Scheme 6 in terms of effective equilibrium constants. Scheme 9 from Rothberg and Magleby (2000) is a subset of Scheme 6. See text for detailed descriptions of all these schemes.

The (mainly) independent voltage and Ca^2+^ activation systems of BK channels can be incorporated into a kinetic model by starting with separate models for each. Scheme 4 in Figure 5 presents a gating mechanism for the voltage-dependent gating of BK channels in the absence of Ca^2+^ (Cox et al., 1997; Cui et al., 1997; Horrigan and Aldrich, 1999; Horrigan et al., 1999), and is similar to models that have been considered for other voltage gated channels (Marks and Jones, 1992; Rios et al., 1993; McCormack et al., 1994). In this 10-state model, each state is comprised of four subunits, as dictated by tetrameric BK channels (Shen et al., 1994). The five states in the upper tier are closed and the five states in the lower tier are open, with an open state indicated by an open circle in the middle of the four subunits on the lower tier. A square subunit indicates that the voltage sensor in that subunit is relaxed (deactivated), whereas a circular subunit indicates that the voltage sensor is activated. The same 10-state Scheme 4 would also apply for voltage dependent gating at 95 μM Ca^2+^ by shading all the squares and circles to indicate that all subunits have bound Ca^2+^ (Shelley et al., 2010). Notice in Scheme 4 that the voltage sensors can activate and deactivate for both open and closed channels, and that there is not an obligatory coupling between voltage sensor movement and channel opening and closing, as channels can open and close with 0, 1, 2, 3, and 4 activated voltage sensors, with Po increasing with an increase in the number of activated voltage sensors (Horrigan and Aldrich, 1999; Horrigan et al., 1999; Rothberg and Magleby, 1999, 2000; Shelley et al., 2010). Further support for Scheme 4 comes from the single-channel observations of Talukder and Aldrich (2000) of multiple components in both open and closed dwell-time distributions at high voltages (+180 to +260 mV) with zero Ca^2+^. They suggest that the multiple components indicate states or combinations of states with different numbers of activated voltage sensors.

Scheme 5 presents a gating mechanism for the Ca^2+^-dependent gating of BK channels (McManus and Magleby, 1991). This scheme parallels the voltage activation shown in Scheme 4 except that activation is by binding Ca^2+^, indicated by a shaded (gray) subunit. Scheme 5 as depicted is for all four voltage sensors activated. The same Scheme 5 with no voltage sensors activated would be indicated by replacing all the round subunits in Scheme 5 with square subunits. Scheme 5 for Ca^2+^-dependent gating has features in common with the Monod, Wyman and Changeux (MWC) model (Changeux and Edelstein, 1998) widely considered for agonist activation of receptor channels. Scheme 5 for Ca^2+^ activation has many of the same general features as Scheme 4 for voltage activation. Both are two-tiered, with five closed states on the upper tier and five open states on the lower tier. There is not an obligatory coupling between Ca^2+^ binding and channel opening and closing, as channels can open and close with 0, 1, 2, 3, and 4 bound Ca^2+^, with Po increasing with the number of bound Ca^2+^ (Magleby and Pallotta, 1983a,b; McManus and Magleby, 1991; Cox et al., 1997; Cui et al., 1997; Rothberg and Magleby, 1998b, 1999, 2000; Horrigan and Aldrich, 2002).

Scheme 6 combines the voltage and Ca^2+^-activation into one model (Rothberg and Magleby, 1999, 2000; Cui and Aldrich, 2000). Scheme 6 includes five repeats of Scheme 4, with 0, 1, 2, 3, and 4 bound Ca^2+^ per channel (left to right). Alternatively, Scheme 6 can be viewed as five repeats of Scheme 5, with 0, 1, 2, 3, and 4 activated voltage sensors per channel (top to bottom). The upper left most tier of one closed and one open state has 0 Ca^2+^ bound and 0 voltage sensors activated. The lower right most tier of one closed and one open state has four bound Ca^2+^ and four voltage sensors activated. Scheme 6 shows that with one Ca^2+^ sensor and one voltage sensor per subunit, the channel could potentially enter 25 closed states on the upper tier and 25 open states on the lower tier during gating, each defined by different combinations of activated voltage sensors and bound Ca^2+^ sites. If the relative positions (adjacent or diagonal) of Ca^2+^ binding and voltage sensor activation on the various subunits in a channel alter the gating, then there would be additional states (Cox et al., 1997; Rothberg and Magleby, 1999; Qian et al., 2006), which will not be considered here. Gating in all 50 states would require changing the Ca^2+^ and voltage over wide ranges. Voltage and Ca^2+^ jointly activate the channel, by shifting the gating from states in the upper left region (low Ca^2+^ and hyperpolarized voltage) of Scheme 6 to states in the lower right region (high Ca^2+^ and large depolarization). For a fixed Ca^2+^ and a fixed voltage, the channel would gate in only a subset of the states in the 50-state scheme.

One highly constrained conceptual method of representing the allosteric action of voltage and Ca^2+^ sensors on the opening and closing transitions between the upper tiers and the lower tiers in Schemes 4–6 can be represented as Scheme 7 in Figure 5, where *n* is the number of voltage sensors activated, *m* is the number of bound Ca^2+^, *H* and *K* are the opening and closing rate constants when *n* and *m* = 0 (no activated voltage or Ca^2+^ sensors), *D* and *E* are the allosteric accelerators for voltage on the opening and closing rate constants, respectively, and *R* and *S* are the allosteric accelerators for bound Ca^2+^ on the opening and closing rates, respectively, (Chen et al., 2012). Scheme 6 with rate constants and expanded to include the allosteric action of both voltage and Ca^2+^ on the opening and closing rates using (Scheme 7) is an extension of earlier models with equilibrium constants (Marks and Jones, 1992; Rios et al., 1993; McCormack et al., 1994; Horrigan and Aldrich, 2002). For Scheme 7, a channel with two voltage sensors activated and three Ca^2+^ bound would have an allosteric acceleration in opening of *D*^2^*R*^3^ and the opening rate would be *HD*^2^*R*^3^.

Scheme 8 represents a clever notation developed by Horrigan and Aldrich (2002) to describe the steady-state Po (equilibrium gating) predicted by the 50-state model in Scheme 6. In Scheme 8, *L*, *J*, and *K* represent the equilibrium constants for the transitions between the closed and open states (C–O), the resting and activated voltage sensors (R–A), and the unbound and bound Ca^2+^ sensors (X–X_Ca2+_), respectively. The four's reflect four voltage and four Ca^2+^ sensors, *D* and *C* are allosteric equilibrium factors for the actions of the voltage sensors and Ca^2+^ sensors on the opening-closing equilibrium, and *E* is a coupling factor for interactions between voltage and Ca^2+^ sensors. Note that the parameters in Scheme 8 have different meanings than those in Scheme 7 and further schemes to be presented in this review. Scheme 8 as shown has equilibrium constants, and consequently gives no information about the time course (kinetics) of gating or single-channel kinetics. Nevertheless, Scheme 8 provides a powerful tool to study gating mechanism of BK channels by indicating the fundamental principles of the gating (Horrigan and Aldrich, 2002), and Scheme 8 can be expanded to use rate constants rather than equilibrium constants, becoming Scheme 6.

## BK channels typically gate in a minimum of 3–4 open states and 5–6 closed states for fixed experimental conditions

**Figure 6:**
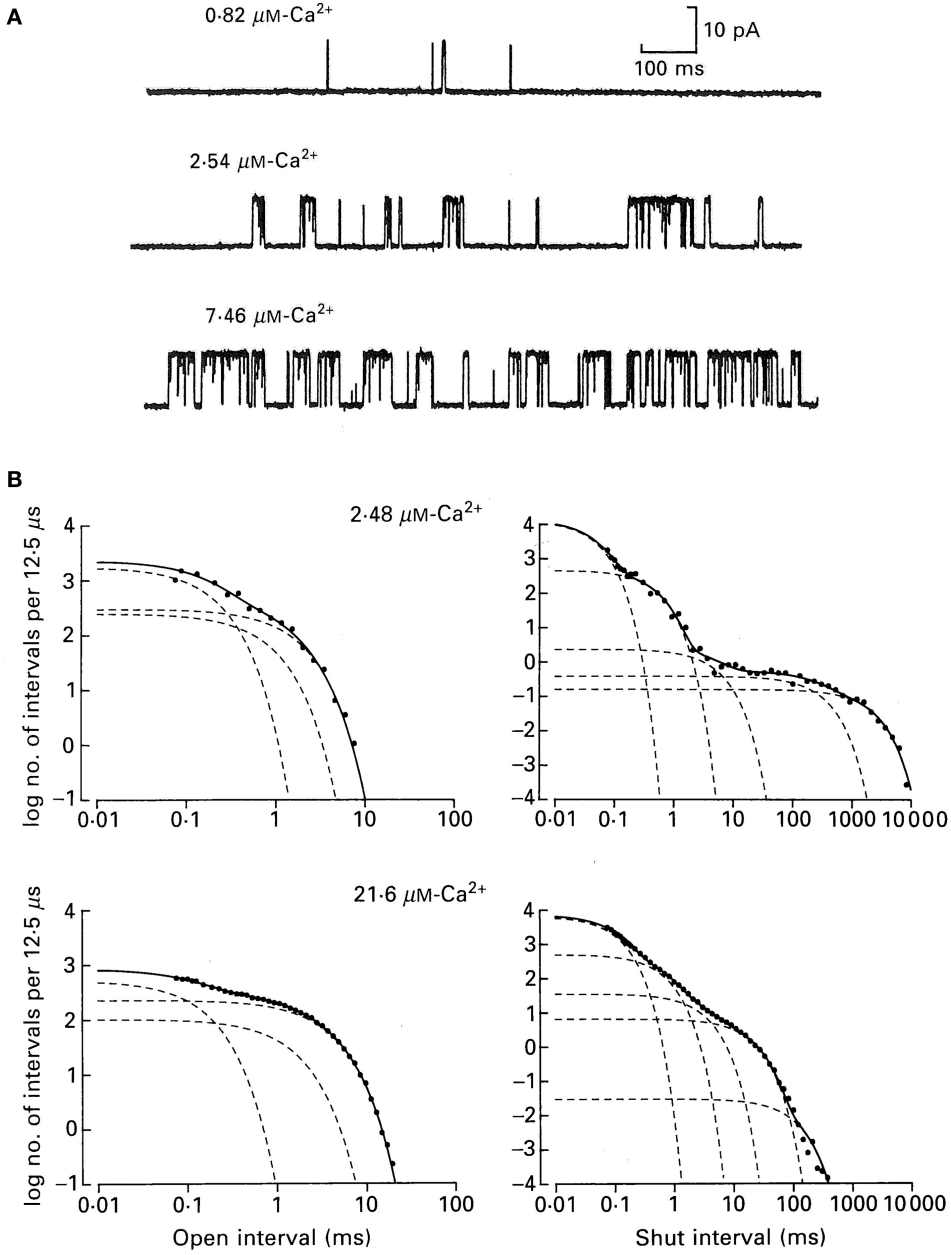
**A BK channel gating in a minimum of three open and five closed states**. **(A)** Single channel records at the indicated Ca^2+^. Opening is upward. **(B)** Open and closed dwell-time distributions at the indicated Ca^2+^ plotted on log–log coordinates for a different channel than those in part **A**. The open distributions were described by the sums of three significant open exponentials (dashed lines summing to make the continuous lines through the data), and the closed distributions were described by the sums of five significant closed exponentials from McManus and Magleby (1991).

Figure 6 shows how the minimum number of kinetic states entered during gating can be estimated from the number of significant exponentials required to describe the dwell-time distributions. Figure 6A presents single-channel recordings obtained from a single BK channel at three different Ca^2+^. The Ca-induced increase in channel activity is readily apparent. Measuring open and closed interval durations from long records of stable data (McManus and Magleby, 1988) and plotting them as frequency histograms on log–log coordinates gave the open (left) and closed (right) dwell-time distributions for the two indicated Ca^2+^ (Figure 6B). These are yet a third type of plot for dwell-time distributions, in addition to those in Figure 4. The ordinate is expressed as log of the number of intervals per 12.5 μs of bin width to correct for the effect of log binning the interval durations, and the abscissa is expressed as the log of the mean duration of the intervals in each log bin. In the log–log plots in Figure 6 each additional exponential is indicated by an inflection in the dwell-time distribution.

From Figure 6B it can be seen that the observed durations of intervals can extend over five orders of magnitude and that the frequencies of intervals can extend over seven orders of magnitude. Such extended information is seldom seen in biology, but is routine for single-channel data because of the huge kinetic space of gating and the fact that frequency (counting) and duration (time) are two physical parameters readily measured by computer with high accuracy.

The open distributions were well-described (continuous line) by the sum of three significant exponential components (dashed lines) and the closed distributions by the sum of five significant exponential components. For experiments of this type for data from single BK channels over a range of Ca^2+^ and voltage (−100 to +100 mV), but with fixed Ca^2+^ and voltage for each determinations, the open distributions were typically described by 3–4 significant open exponential components and the closed distributions by 4–6 significant closed exponential components, suggesting gating in a minimum of 3–4 open states and 4–6 closed states (McManus and Magleby, 1988, 1989, 1991; Rothberg and Magleby, 1999). Detecting 3–4 open states and 4–6 closed states appear more consistent with the 10-state Schemes 4 and 5 than the 50-state Scheme 6. If the 50-state Scheme 6 represents the proposed gating mechanism, then why aren't 25 significant closed and 25 significant open exponentials detected, one for each of the 50 states? One reason presented earlier is that the 50 states are potential states based on the modular structure of BK channels. For any given Ca^2+^ and voltage, the gating would be effectively limited to only a small subset of the 50 states, so only that subset would generate exponentials with sufficiently large areas to detect. Furthermore, calculations on large multistate models show that many of the expected time constants for 50-state models can be nearly identical or too close to detect. It will be shown in sections below, that Ca^2+^ dependent gating at fixed voltage, and voltage dependent gating at fixed Ca^2+^, can each be approximated by the 10-state Schemes 5 and 4, respectively, consistent with the numbers of significant detected exponential components.

The observation of typically 5–10 significant exponentials in the dwell-time distributions of BK channels (Magleby and Pallotta, 1983b; McManus and Magleby, 1988) providing experimental evidence for gating in a minimum of 5–10 states, was met with skepticism by many back at the time, even though it had been previously proposed for decades that agonist activated channels and also voltage activated channels would be expected to gate in 5–10 or more states (Hodgkin and Huxley, 1952; Monod et al., 1965; Nonner, 1980). To avoid the need for large numbers of states, even though expected, the much simpler fractal model gained interest until it was shown to be inconsistent with the experimental data for four different ion channels (McManus et al., 1988).

## Inverse relationship between the durations of adjacent open and closed intervals

**Figure 7:**
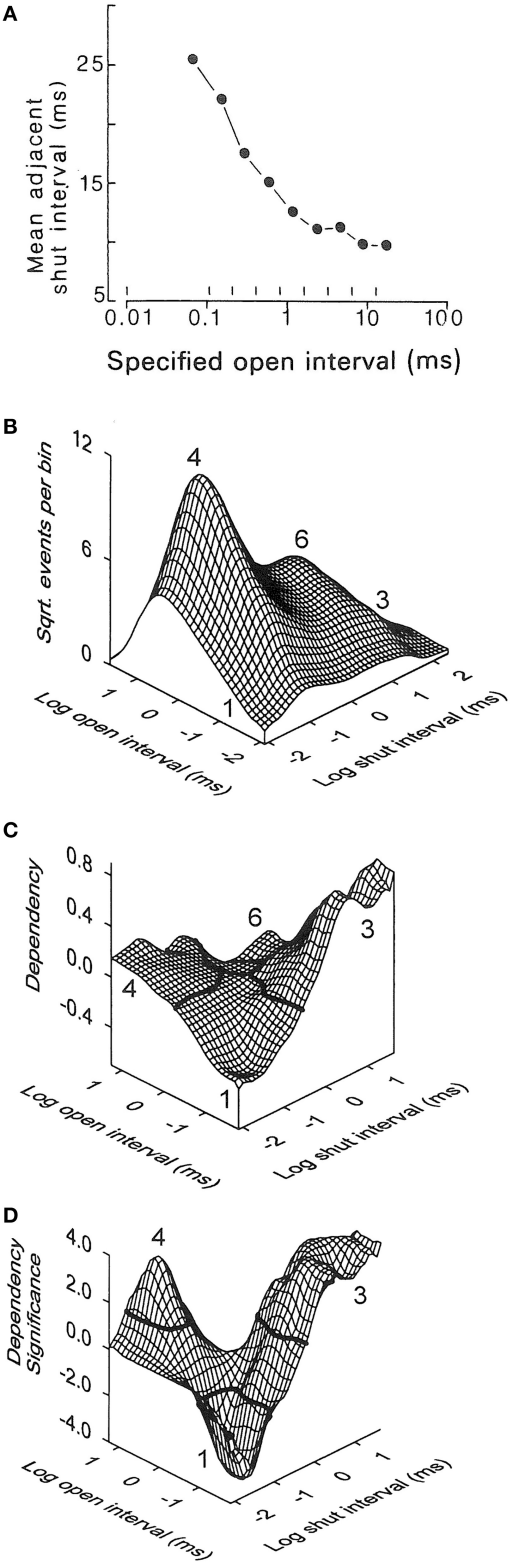
**The inverse relationship between the durations of adjacent open and closed intervals suggests two-tiered gating with the lifetimes of the open states on the lower tier inversely related to the lifetimes of the closed states above them on the upper tier. (A)** Inverse correlation between the mean durations of closed intervals adjacent to open intervals of specified durations delineated by the vertical lines near the abscissa. 0.7 μM Ca^2+^, +30 mV, 64,000 open and closed intervals were analyzed (from McManus et al., 1985). **(B-D)** Kinetic structure of a BK channel details the inverse relationship between durations of adjacent open and closed intervals. **(B)** 2D dwell-time distribution of adjacent open and closed interval pairs presented in a Sigworth and Sine transformation. The *x* and *y* axes indicate the logs of the means of the adjacent open and closed interval durations in each 2D bin. The ordinate gives the square root of the number of adjacent interval pairs in each 2D bin defined by the *x* and *y* axis. The greatest frequency is at number 4 for longer openings adjacent to briefer closings (12.3 μM Ca^2+^, +30 mV). **(C)** Dependency plot of the excess frequency of interval pairs over that expected if open and closed intervals pair at random. There were 45% more longer closed intervals adjacent to briefer open intervals (number 3), 15% more longer open intervals adjacent to briefer closed intervals (number 4), and 40% fewer briefer open intervals adjacent to briefer closed intervals (number 1), than would be expected by chance pairing. Dependency for any given bin is defined as the difference between the observed number of intervals in that bin minus the number of intervals expected for independent pairing, divided by the number expected for independent pairing. **(D)** The excesses of paired intervals in part **C** are significant (*P* < 0.05) for interval pairs above the solid black lines at numbers 3 and 4, and the deficits are significant for interval pairs below the solid black line at number 1. A significant deficit of longer open intervals adjacent to longer closed intervals (at number 6) cannot be seen on the back side of the plot. 28,560 intervals were analyzed (from Rothberg and Magleby, 1998b). The observed inverse relationships between the durations of adjacent open and closed interval pairs would be consistent with Schemes 4–6 if the closed state lifetimes in these schemes progressively decreased from left to right, and the open state lifetimes progressively increased from left to right.

General information about gating mechanisms can be obtained from the relationships between adjacent open and closed interval durations (McManus et al., 1985; Colquhoun and Hawkes, 1987; Magleby and Song, 1992; Rothberg and Magleby, 1998b). To generate correlations there needs to be more than one gateway state in the kinetic scheme, where the number of gateway states in a kinetic scheme is given by the minimal number of states that have to be removed to sever all connections between open and closed states (Colquhoun and Hawkes, 1987). For Schemes 1 and 3 there would be no correlation between the durations of adjacent open and closed intervals because there is only one gateway state connecting open and closed states. In Schemes 2, 4, 5, and 6, there are 2, 5, 5, and 25 gateways between open and closed states so that these schemes could show correlations between adjacent open and closed interval durations. To test for correlations, McManus et al. (1985) plotted the mean durations of open intervals adjacent to specified ranges of closed intervals (Figure 7A). There was an inverse relationship between the durations of adjacent open and closed intervals. This inverse relationship can be observed directly in the single-channel current records in Figure 6A, where brief open intervals are typically adjacent to longer closed intervals and longer open intervals are typically adjacent to briefer closed intervals.

A more rigorous demonstration of this inverse relationship for essentially all possible pairs of adjacent open and closed interval durations, including showing the significance of the inverse relationship, is presented in Figures 7B–D (Rothberg and Magleby, 1998b). Part **B** shows 2D dwell-time distributions of all theoretically possible pairs of adjacent interval durations, plotted in the Sigworth and Sine transform. Part **C** shows a dependency plot of the data in part **B** which indicates which pairs of adjacent open and closed intervals are in excess and which are in deficit over that expected by chance pairing alone. Part **D** presents a dependency significance plot of the data in part C indicating the significance of the excesses and deficits of intervals. (These plots are described in-depth in the legend of Figure 7). Plots **C** and **D** would be a flat plane at 0.0 if adjacent open and closed intervals paired by chance alone, as would be the case if there were only one gateway state. The significant correlations rule out gating mechanisms like Scheme 3, with one gateway state between open and closed intervals. The observed inverse correlations between adjacent open and closed interval durations suggest gating mechanisms like Schemes 4–6 in which the lifetimes of the closed states on the upper tiers progressively decrease and the lifetimes of the open states on the lower tiers progressively increase as more voltage sensors are activated and/or more Ca^2+^ is bound. For a graphical demonstration of this concept, see Magleby and Song (1992).

## Gating of BK channels is consistent with microscopic reversibility

To develop a gating mechanism for an ion channel it is necessary to know whether there is an external energy source driving the gating. In an excised patch of membrane held at a moderate voltage such as +30 mV, BK channels can gate with stable kinetics for many hundreds of thousands of open and closed transitions over tens of minutes. Where is the energy source for the gating? In order to record single channel currents there has to be an electrochemical (voltage and/or concentration) gradient to drive K^+^ through the channel. If this energy source provided energy for gating, then the single-channel kinetics for the same current record analyzed in the forwards and backwards directions could differ (Steinberg, 1987; Song and Magleby, 1994; Colquhoun and Hawkes, 1995b). No differences were found in single-channel records analyzed in the forwards and backwards directions for BK channels (Song and Magleby, 1994), indicating that the gating for BK channels was consistent with microscopic reversibility. Consequently when fitting data for BK channels, microscopic reversibility is maintained by keeping the product of the rate constants in one direction around each loop of states equal to the product of the rate constants in the opposite direction around the same loop of states for every loop in the model (Colquhoun and Hawkes, 1995b). For Scheme 2 the product of *k*_3–1_ × *k*_1–2_ × *k*_2–4_ × *k*_4–3_ would need to equal the product of *k*_1–3_ × *k*_3–4_ × *k*_4–2_ × *k*_2–1_. Hence one of the rate constants in each loop when fitting is not free but is determined by the other seven.

## Activation of BK channels by Ca^2+^

**Figure 8:**
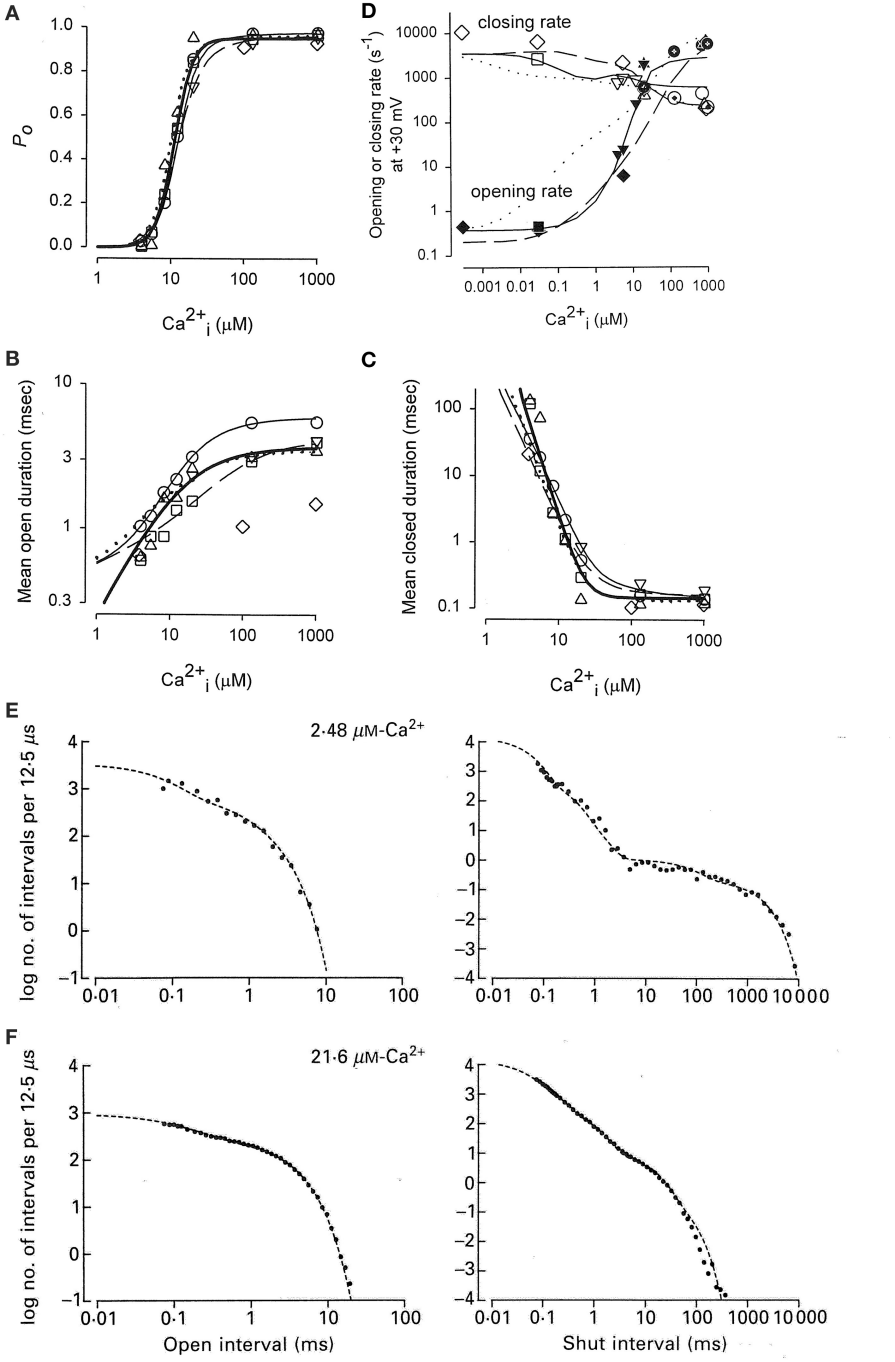
**Accounting for the Ca^2+^ dependent gating of BK channels with Scheme 5**. **(A)** Ca^2+^ activates BK channels with a Hill coefficient of 3.5 (heavy black line), indicating cooperative action of Ca^2+^ in opening the channel. **(B)** Ca^2+^ increases mean open interval duration with a Hill coefficient of 1.0 (thick black lines) and **(C)** decreases mean closed interval duration with a Hill coefficient of −3.5. **(D)** Effective mean opening and closing rates as a function of Ca^2+^. The major action of Ca^2+^ binding is to cooperatively increase the opening rate. The thin lines are the description of the data for models based on subsets of the states in Scheme 6 (Rothberg and Magleby, 1999, 2000). **(E,F)** Predicted dwell-time distributions for Scheme 5 with the two lower left-most open states not included, as they were entered too infrequently to alter the likelihood of the model. Parts **A–C** are from Rothberg and Magleby (1999). Part **D** is from Rothberg and Magleby (2000). Parts **E** and **F** are from McManus and Magleby (1991). All data at +30 mV.

Activation of a BK channel by Ca^2+^ at the single-channel level was shown in Figure 6A. Data obtained from the analysis of four different patches, each containing a single BK channel, are shown in Figure 8A (Rothberg and Magleby, 1999). (All data in Figure 8 were collected at +30 mV.) There was a steep dependence of Po on Ca^2+^_i_, with a maximal Po of 0.95, a half activation, K_0.5_, at 11.1 μM Ca^2+^_i_, and a Hill coefficient of 3.5, (thick line from fitting the Hill equation), suggesting four or more bound Ca^2+^ are required for maximal activation of the channel. Note that four bound Ca^2+^ would be consistent with Schemes 5 and 6. The Hill coefficient of 3.5 indicates a cooperative action of Ca^2+^ in activating the channel, with at least four sites bound for full activation. Niu and Magleby (2002) found that decreasing the number of Ca^2+^ bowls (by mutation) decreased the Hill coefficient, with Hill coefficients of 4.1, 3.5, 2.6, 1.8, and 1.4 observed for 4, 3, 2, 1, and 0 functional Ca^2+^ bowls. Their observations reveal directly that BK channels can gate with 0–4 high affinity allosteric Ca^2+^ bowl activators. This would be consistent with the two-tiered Schemes 5 and 6 where the channel can open with 0–4 Ca^2+^ sensors bound. Niu and Magleby (2002) also found that a model with 0–4 Ca^2+^ bowl sensors together with four RCK1 Ca^2+^ sensors could describe the Po vs. Ca^2+^ and Hill slope data.

Increasing Ca^2+^ increased observed mean open interval duration 10-fold with a Hill coefficient of 1.02 (Figure 8B) and decreased observed mean closed interval duration about 1000-fold with a Hill coefficient of −3.48 (thick lines), for changes in Ca^2+^ from 3 to 1000 μM (Figure 8C). Determining the effective mean opening and closing rate constants from the inverse of the mean closed and open dwell-times (Equations 3 and 4) over a wider range of Ca^2+^ gave the plots in Figure 8D, where increasing Ca^2+^ from <0.1 to 1000 μM increased the effective opening rate constant 10,000-fold and decreased the effective closing rate constant about 30-fold. Hence, the major action of Ca^2+^ is a highly cooperative increase in the effective rate constant for opening (leaving the closed states), indicating destabilization of the closed states, with a much smaller increase of slowing the effective rate constant for leaving the open states (closing), which gives a much smaller stabilization of the open states.

To explore what types of gating mechanisms could describe the Ca^2+^ activation of BK channels, McManus and Magleby (1991) tested 11 models that were subsets of Scheme 5 and also an extension of Scheme 3 with five closed and four open states with one gateway state. They collected single channel data at 3–4 different Ca^2+^ for each of five patches, each containing a single BK channel. Typically >100,000 intervals were recorded from each channel. All data were collected at +30 mV. Simultaneous maximum likelihood fitting of the data for each channel then gave the most likely estimates for the rate constants and likelihood values. Ranking of the models then indicated that Scheme 5 gave excellent descriptions of the single-channel kinetics and Ca^2+^ activation of the channels. For this channel the two left most open states on the lower tier did not improve the likelihood so they were omitted, consistent with the detection of only three significant open states for this channel. This eight-state Scheme 5 could describe the Ca^2+^ dependence of the single channel kinetics (Figures 8E,F). The Po vs. Ca^2+^ curve was also well-described and the correlations between open and closed interval durations was approximated (not shown). The principle activation–deactivation pathway was C_0Ca_-C_1Ca_-C_2Ca_-C_3Ca_-O_3Ca_-O_4Ca_. The closed states C_0Ca_-C_1Ca_-C_2Ca_-C_3Ca_ greatly decreased their lifetimes with each successive Ca^2+^ binding, and the open states O_2Ca_-O_3Ca_-O_4Ca_ moderately increased their lifetimes.

Adding an intermediate tier of brief closed states (flickers) significantly improved the description of the Ca-dependent gating (Rothberg and Magleby, 1999). More recently, such intermediate flicker states, termed flip states (Lape et al., 2008) or intermediate states (Mukhtasimova et al., 2009) have been described in the gating of nicotinic receptor channels. Thus, Scheme 5 gave reasonable descriptions of the Ca^2+^-dependence of the single-channel kinetics for a fixed voltage, with the addition of a tier of flicker closed states improving the description of the data.

## Activation of BK channels by voltage

**Figure 9:**
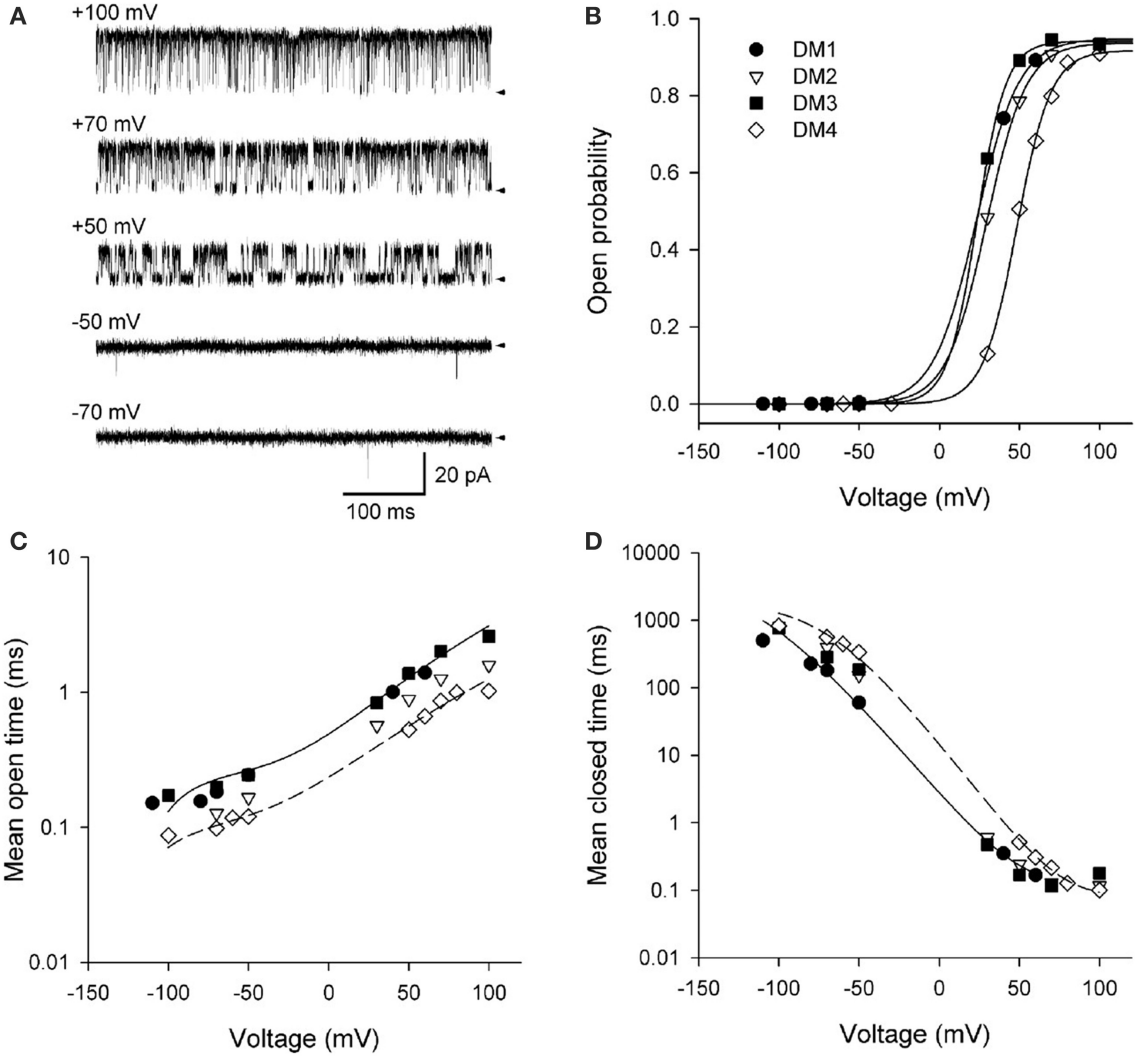
**Depolarization activates BK channels**. **(A)** Single-channel records at the indicated voltages. Depolarization activates BK channels, as indicated by the increased time spent open with increasing depolarization. The arrows indicate the closed current level. The channel has the high affinity RCK1 Ca^2+^ site (D362A/D367A) and the low affinity Mg^2+^ site (E399A) on each subunit removed by mutation (Xia et al., 2002). Ninety five micromolar Ca^2+^ was used to saturate the Ca^2+^ bowls. **(B)** Po vs. voltage plots for four different single BK channels. The lines are Boltzman fits indicating an e-fold (2.72) increase in Po for each additional 11.4 mV of depolarization. **(C,D)** Depolarization from −100 to +100 mV increased mean open time 12-fold and decreased mean closed time 4500-fold. The thin lines are the predicted response for DM1 (continuous lines) and DM4 (dashed lines) with Scheme 12. Figures are from Shelley et al. (2010).

Shelley et al. (2010) studied the activation of BK channels by voltage at the single-channel level. The activation of a single BK channel by voltage is shown in Figure 9A for voltages ranging from −70 to +100 mV with Ca^2+^ fixed at 95 μM Ca^2+^. Po ranged from 2 × 10^−4^ at −70 mV to 0.94 at +100 mV. The fixed 95 μM Ca^2+^ was used to left shift the activation of the channel to voltage ranges where the channel could be fully activated (0.95) without the need to apply such high voltages that the membrane would be destroyed for the longer duration recordings required for single-channel analysis. The RCK1 Ca^2+^ site and the Mg^2+^ site were removed by the mutations D362A/D367A and E399A, respectively (Shi et al., 2002; Xia et al., 2002; Zeng et al., 2005), leaving the Ca-bowl, which would be fully saturated with the 95 μM Ca^2+^, more than 20 times the apparent K_D_ (Bao et al., 2002; Xia et al., 2002). The saturating Ca^2+^ would shift the gating to the 10-state model described by the right most 10-states in Scheme 6. This Ca^2+^ saturated 10-state scheme would be indicated by filling in all of the subunits in Scheme 4 to indicate that each subunit has a bound Ca^2+^.

Data from four different patches, each containing a single channel is shown in Figures 9B–D. Fitting the Po vs. voltage data with a Boltzman equation in Figure 9B indicated an 11.4 mV depolarization for an e-fold (2.7 times) increase in Po with an effective partial charge movement across the electric field of the membrane of 2.3 units of elementary charge (e_o_) for activation (Shelley et al., 2010). This can be compared to Shaker K_V_ channels, which are more voltage sensitive, with a 2.4 mV depolarization for an e-fold change in Po and an effective partial charge movement of 12.3 e_o_ for activation (Schoppa et al., 1992). The decreased voltage sensitivity of BK channels arises because only the lowest of the four arginines in S4 of each voltage sensor contributes to the voltage dependence of BK (Ma et al., 2006). Changing the voltage from −100 to +100 mV decreased the observed mean closed time 4500-fold (Figure 9D), while increasing the observed mean open time 12-fold (Figure 9C). Hence, the predominant action of depolarization is to destabilize the closed states, with a much smaller action on stabilizing the open states.

**Figure 10:**
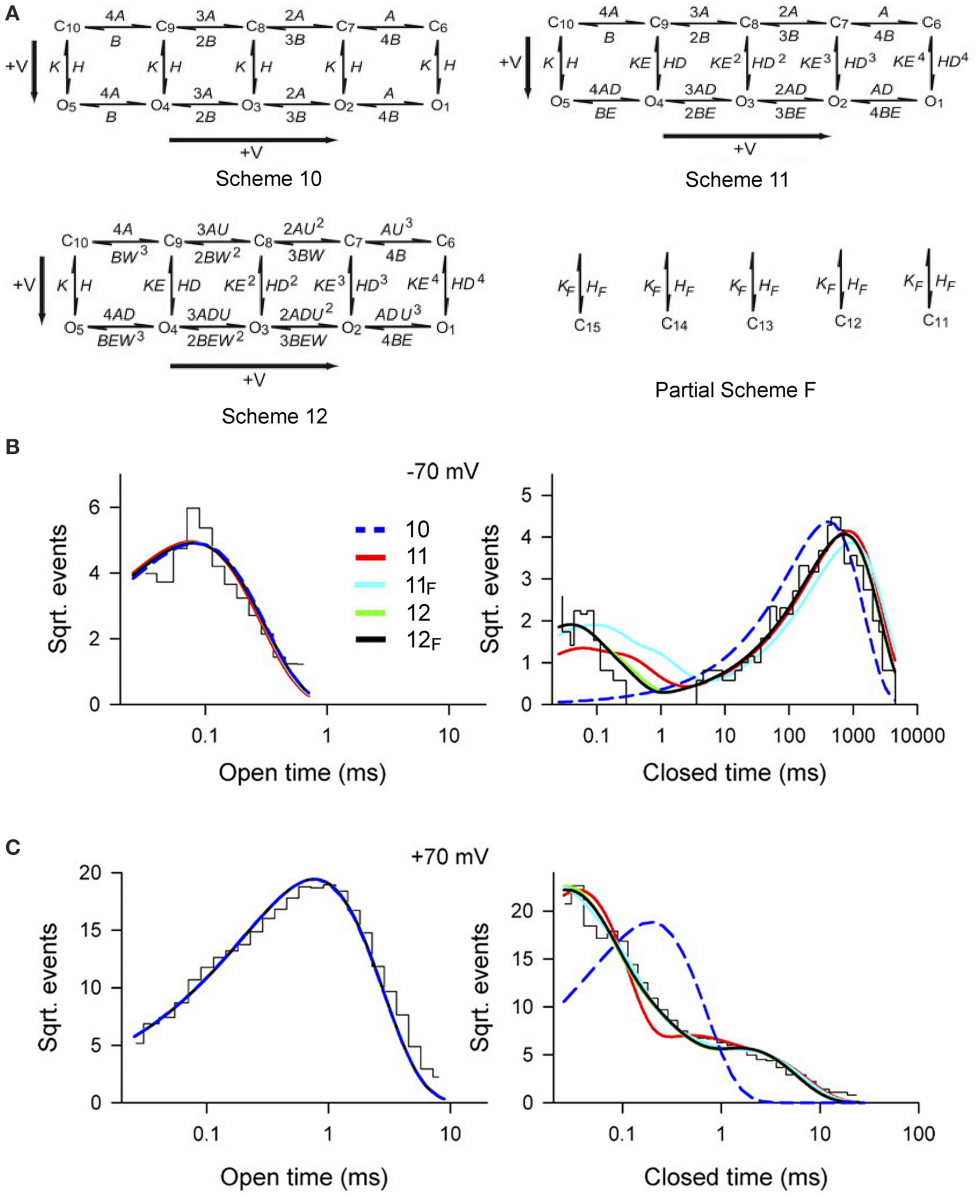
**Accounting for the voltage dependent gating of BK channels with Scheme 4, as detailed by Schemes 11 and 12. (A)** Examined gating mechanisms. See text for details. **(B,C)** Open and closed dwell-time distributions at −70 and +70 mV, as indicated, for experimental conditions as specified in Figure 9. The various lines are the predicted descriptions for the various examined schemes, as indicated. Schemes 12_F_ and 12 gave excellent descriptions of the data. Schemes 11_F_ and 11 approximated the data. Scheme 10 could be rejected outright. Predictions of all the examined schemes essentially superimposed for the open distributions, and Schemes 12 and 12_F_ typically superimposed for the closed distributions. Figures from Shelley et al. (2010).

To examine whether Scheme 4 could account for the voltage activation at the single-channel level, specific Schemes 10, 11, and 12 (Figure 10A) were examined by Shelley et al. (2010). Rate constants *A* and *B* for voltage sensor activation and deactivation, respectively, and rate constants *H* and *K* for channel opening and closing, respectively, were exponentially dependent on voltage such that

(8) $$Rate(V)=Rate(V_{\text{o}})\text{exp}(\text{qV/eo}25.5\text{ mV})$$

where voltage (V) is in mV, V_o_ is 0 mV, and q is partial charge displacement in units of e_o_ for the conformational change described by the rate constant, and 25.5 mV (at 23°C) indicates that the rate constant will change e-fold for each elementary unit of charge moved through 25.5 mV of membrane potential (Hille, 2001). For example, for q of 0.3 e_o_ and Rate (V_o_) = 100/s, then the rates at −100 and +100 mV would be 30.8/s and 324/s. The 4, 3, 2, and implied 1 preceding each rate constant above the forward arrows indicates the number of subunits with deactivated voltage sensors, and the implied 1 and 2, 3, and 4 preceding each rate constant below the backwards arrows indicates the number of subunits with activated voltage sensors. Partial Scheme *F* would add a tier of flicker closed states beneath the other schemes with subscript *F* added to the scheme designation.

Single-channel data collected over a range of voltages were simultaneously fit to each of the kinetic Schemes in Figure 10A. Scheme 12_F_ (with the tier of flicker closed states) ranked first followed by Schemes 12, 11_F_, 11, and 10. Schemes 12_F_ and 12 gave excellent description of the voltage dependence of the distributions (Figures 10B,C). As different as these Sigworth and Sine plots may look from the log–log plots for Ca^2+^ activation in Figures 8E,F, if the same transforms were used for the plots, those with the same Po would look relatively similar. Scheme 10 did not describe the closed dwell-time distributions or the shift in the closed dwell-time distribution with voltage and can be rejected outright. Predictions of all the schemes superimposed for the open distributions and also for Schemes 12 and 12_F_ for the closed distributions. Interestingly, all of the schemes including Scheme 10 gave excellent descriptions of the Po vs. V curve (equivalent to the continuous lines in Figure 9B), indicating that the ability of a model to describe Po vs. voltage is not a critical test of gating mechanism.

For Schemes 12_F_ and 12 there was cooperativity (*U*) in subunit activation with activation of deactivated voltage sensors about *U* = 4.5 times faster for every activated S4, with little cooperativity in subunit deactivation (*W* = 1.3). Whether there is actual cooperativity in gating or whether the cooperativity is just a product of the idealized relationships between rate constants in Scheme 12_F_ and 12 will require additional experiments to resolve. In these schemes, the major voltage dependence of the gating arises from two separate steps. In the first step there is a voltage dependent increase in the rate of voltage sensor activation (mean q_A_ = 0.34 e_o_) and a voltage dependent slowing of voltage sensor deactivation (mean q_B_ = −0.18 e_o_), where q is the partial charge movement associated with these conformations. In the second step this voltage dependent change in the number of activated voltage sensors is then allosterically coupled to the opening and closing transitions in Schemes 12_F_, 12, 11_F_, and 11 through the coupling factors *D* and *E*. Each activated voltage sensor changes the opening rate *D*-fold and the closing rate *E*-fold. The allosteric acceleration in opening rate *D* for each activated voltage sensor was 22.9, 28.3, 17.9, 17.4 for Schemes 11, 11_F_, 12, and 12_F_, respectively, and the allosteric acceleration in closing rate, *E*, ranged from 1 to 1.4 for channel closing. A value of *D* of 20 would indicate a 20-, 400-, 8000-, and 160,000-fold increases in opening rate for 1–4 activated voltage sensors, respectively, compared with little effect on the closing rate. Hence, depolarization activates predominantly by accelerating the opening rate, destabilizing the closed states.

A smaller voltage dependence of increasing the opening rate constant *H* with depolarization (q_H_ ~0.2 e_o_) and decreasing the channel closing rate constant *K* (q_K_ ~ -0.2 e_o_) was also observed (Shelley et al., 2010). The voltage dependence of opening *H* and closing *K* rate constants is thought to reflect the further movement of the S4 voltage sensors as the channels open or close (Horrigan and Aldrich, 1999, 2002; Horrigan et al., 1999). To a first approximation, the estimates of partial charge determined from the single-channel analysis (Rothberg and Magleby, 2000; Shelley et al., 2010) are consistent with those estimated from gating current and macro current analysis following voltage steps (Horrigan and Aldrich, 1999, 2002; Horrigan et al., 1999).

The data of Shelley et al. (2010) then show that voltage activation of BK channels can be described at the single-channel level by the 10-state two tiered Scheme 4 as detailed in Scheme 12 (and approximated by Scheme 11), and that an added tier of flicker closed states (Partial Scheme F) improves the description of the data (Figures 10B,C). The voltage dependence arises predominantly from each activated voltage sensor greatly accelerating the opening rate.

## Synergistic activation of BK channels by Ca^2+^ and voltage

**Figure 11:**
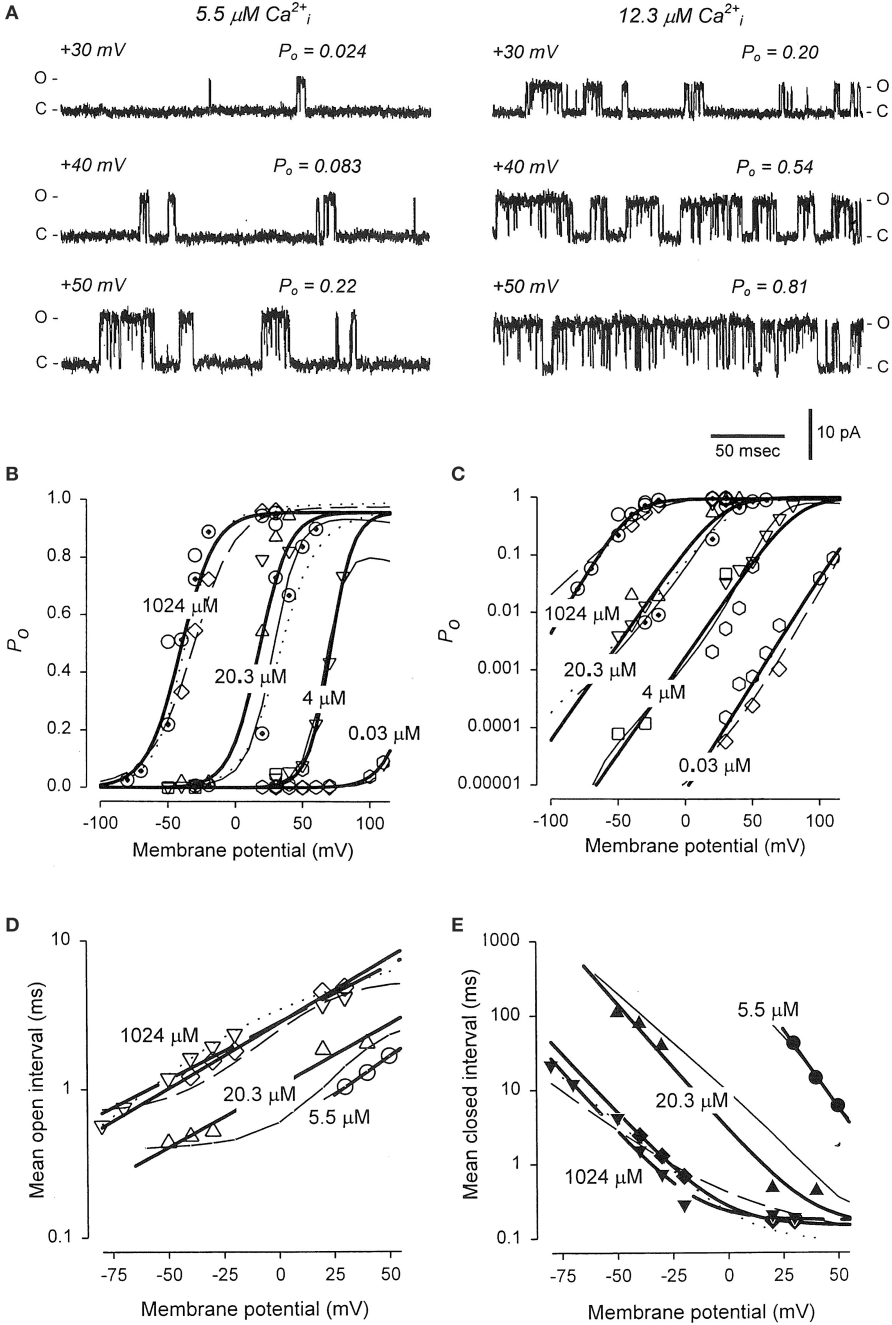
**Joint activation of BK channels by Ca^2+^ and depolarization**. **(A)** Single-channel records showing that increasing Ca^2+^ or depolarization increased Po, and increasing both together gave a synergistic increase in Po. Currents for six different combinations of Ca^2+^ and voltage are shown. Open (o) and closed (c) current levels are indicated. **(B,C)** Linear and semi-logarithmic plots of Po vs. membrane potential for seven different single-channel patches at four different Ca^2+^. Thick solid lines are the mean response. Increasing Ca^2+^ left shifts the plots to more negative potentials, indicating that less depolarization is required to activate the channel as Ca^2+^ is increased. **(D,E)** The predominant action of Ca^2+^ and voltage is to decrease mean closed interval duration (part **E**), with a much smaller effect on increasing mean open interval duration (part **D**), and this is the case over a range of Ca^2+^ and voltage. Thick solid lines are the mean response. The thin continuous, dotted, and dashed lines are the predicted responses with Scheme 9, a subset of the 50 state Scheme 6. Figures from Rothberg and Magleby (2000).

The previous sections showed that Schemes 4 and 5 could account separately for the voltage and Ca^2+^ activation of BK channels at the single-channel level. Combining these two schemes gives the 50-state model in Scheme 6. This section reviews the work of Rothberg and Magleby (2000) showing that Scheme 6 can account for the synergistic activation of BK channels by Ca^2+^ and voltage. The joint activation of a BK channel by voltage and Ca^2+^ is shown in Figure 11. Increasing either Ca^2+^ (left to right comparison) or depolarization (upper to lower comparison) increased Po. Jointly increasing Ca^2+^ and depolarization (diagonal from upper left to lower right) led to synergistic increases in Po. Po vs. voltage plots for data from seven single-channel patches are plotted over a range of voltage and Ca^2+^ in Figure 11B on linear (B) and semi-logarithmic (C) coordinates. The thick solid lines are Boltzman fits, indicating an effecting partial charge movement of 2.3 e_o_ in determining the Po, which was little affected by Ca^2+^, as indicated by the near parallel shifts (Figures 11B,C). Further support that Ca^2+^ has little effect on the charge movement is the observation by Shelley et al. (2010) that with 95 μM Ca^2+^ their estimate of the partial charge movement was the same as in Figures 11B,C. Increasing Ca^2+^ gave approximately parallel left shifts in the Po vs. voltage curves, suggesting independent activation by Ca^2+^ and voltage (Horrigan and Aldrich, 2002), consistent with the modular model of separate voltage and Ca^2+^ activation systems. For essentially zero Ca^2+^ (0.001 μM), a voltage of +150 mV was required to half activate the channel. With 20.3 μM Ca^2+^ the voltage required to half activate the channel was +15 mV, for a left shift of −135 mV by 20.3 μM Ca^2+^. For BK channels, facilitation or inhibition of activation by various agents or mutations is often quantified in terms of the equivalent left or right shifts in the voltage, respectively, required to restore half activation.

As was shown in the previous sections for Ca^2+^ activation (Figures 8B–D) and voltage activation (Figures 9C,D) acting separately, activation over a range of Ca^2+^ and voltage also occurs predominately by decreasing closed interval duration with a smaller increase in open interval duration (Figures 11D,E). With increased Ca^2+^ and depolarization, the mean closed interval durations decreased to an apparent steady-state value of about 0.18 ms, reflecting mainly the briefest closed intervals (the flickers within bursts) that dominate at high Po. The reciprocals of the thick lines in Figures 11D,E indicate a partial charge movement for channel closing of −0.5 e_o_ and for channel opening of 1.8 e_o_. Summing these and changing the sign for channel closing to correcting for the fact that slowing channel closing increases Po, gives a net partial charge for channel activation of 2.3 e_o_, as determined from the Po vs. voltage plots in Figures 11B,C. These observations indicate that the major effect of depolarization on Po is through an increase in the channel opening rate.

**Figure 12:**
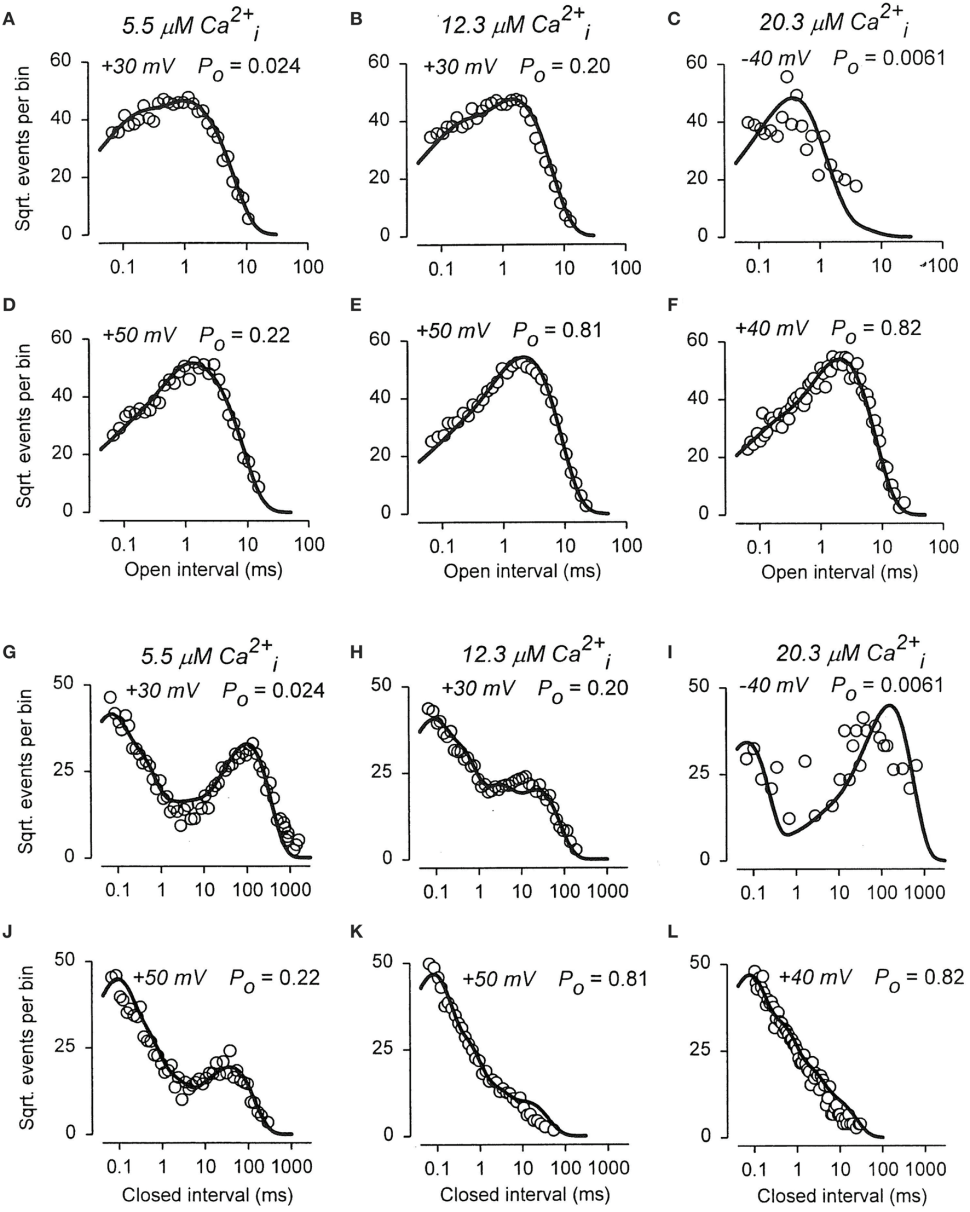
**The joint Ca^2+^ and voltage dependent gating of BK channels is consistent with a 50 state two-tiered gating mechanism. (A–L)** Open and closed dwell-time distributions presented in the Sigworth and Sine transform are plotted for six different combinations of Ca^2+^ and voltage. The heavy lines are the predicted distributions with Scheme 9 with a single set of gating parameters. Because Scheme 9 is a subset of the 50 state Scheme 6, then Scheme 6 should describe the data equivalent or better than Scheme 9. Figure from Rothberg and Magleby (2000).

To examine whether the 50-state Scheme 6 that combines Ca^2+^ and voltage activation could account for the joint activation of BK channels by Ca^2+^ and voltage, Rothberg and Magleby (2000) determined whether Scheme 9, a subset of the 50-state scheme, could account for the joint activation. The rational for the reduced state scheme was two-fold: (1) if the reduced state scheme could account for the single-channel kinetics, then the full 50-state scheme would also be able to account for the kinetics because Scheme 9 is contained within the 50-state scheme; and (2) the reduced state scheme decreased the numbers of rate constants sufficiently that they could be determined through simultaneous fitting of data obtained over a range of Ca^2+^ and voltage. Figure 12 presents experimental open and closed dwell-time distributions for six different combinations of Ca^2+^ and voltage with Po ranging from 0.0061 to 0.82. The reduced state Scheme 9 gave good to excellent descriptions of the data (thick lines). Hence, the more complex 50-state scheme would also be able to account for the single-channel kinetics equivalently or better. Chen et al. (2012) made a direct test of the 50-state Scheme 6 using Scheme 7 to constrain rate constants to greatly reduce the number of free parameters, and found that the highly constrained model could approximate the description of the data, and that the addition of a tier of flicker closed states improved the likelihood of the description. Hence, the 50 state Scheme 6 is consistent with the voltage and Ca^2+^ dependent activation of the BK channel.

## Accounting for the bursting behavior of BK channels

**Figure 13:**
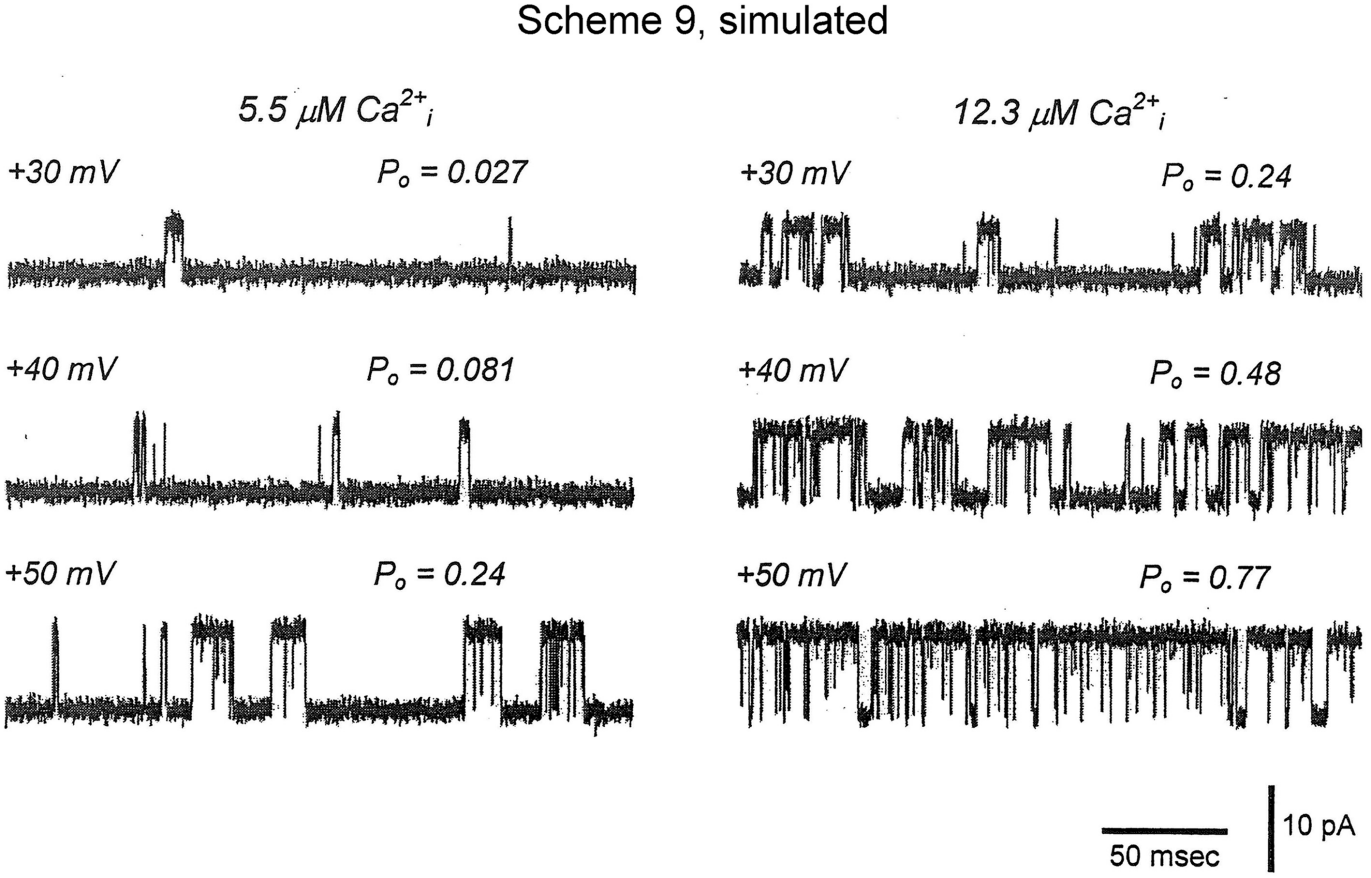
**Accounting for the bursting behavior of BK channels with Schemes 9 and 6**. Simulated single-channel currents from Figure S1 of Rothberg and Magleby (2000) for comparison to the experimental data in Figure 11A. The currents were simulated with Scheme 9 with filtering and noise equivalent to that of the experimental data. The values of Po were obtained from 10,000 simulated intervals at each of the indicated Ca^2+^ and voltage. Opening is upward. Scheme 9 predicts single-channel Po and bursting behavior records that are remarkably similar to the experimental data. Because Scheme 9 is a subset of Scheme 6, then Scheme 6 would also be able to predict the single-channel data equivalently or better.

Inspection of the single-channel records in Figures 1, 6, 9, 11 show that gating of BK channels (as with most other channels) occurs in bursts of openings, with each burst comprised of typically longer openings separated by briefer closings (flickers). The bursts are then separated from each other by longer duration closings (Magleby and Pallotta, 1983a; Nimigean and Magleby, 1999, 2000). To examine whether Scheme 9 captures the single-channel bursting behavior, single-channel records predicted by Scheme 9 were simulated with filtering and noise and plotted in Figure 13 (From Figure S1 in Rothberg and Magleby, 2000). Comparison of the experimental single-channel current records (Figure 11A) with the predicted single-channel records (Figure 13) shows that Scheme 9, and consequently Scheme 6, can generate single-channel current records with bursting behavior that mimics the experimental data.

## Functions of the gating ring (CTD)

As indicated previously in the Modular Structure Section, extensive mutational studies have suggested two high affinity Ca^2+^ binding sites on each subunit of BK channels, the Ca-bowl and the RCK1 site, located in the RCK2 and RCK1 domains, respectively, of each of the four subunits that form the gating ring. Extensive mutational studies also indicated that a low affinity Mg^2+^ site is sandwiched between the top of the gating ring and the cytosolic side of each of the voltage sensors (see Figure 2 and associated references). On this basis, removing the gating ring should remove all of the Ca^2+^ and Mg^2+^ sensitivity. Budelli et al. (2013) found that removing the 827 amino acid gating ring and replacing it with a short 11 amino acid tail removed all of the Ca^2+^ and Mg^2+^ sensitivity from the BK channel. Hence, as indicated in the modular diagram in Figure 2, the Ca^2+^ sensors are located in the gating ring and the Mg^2+^ sensors require the gating ring.

**Figure 14:**
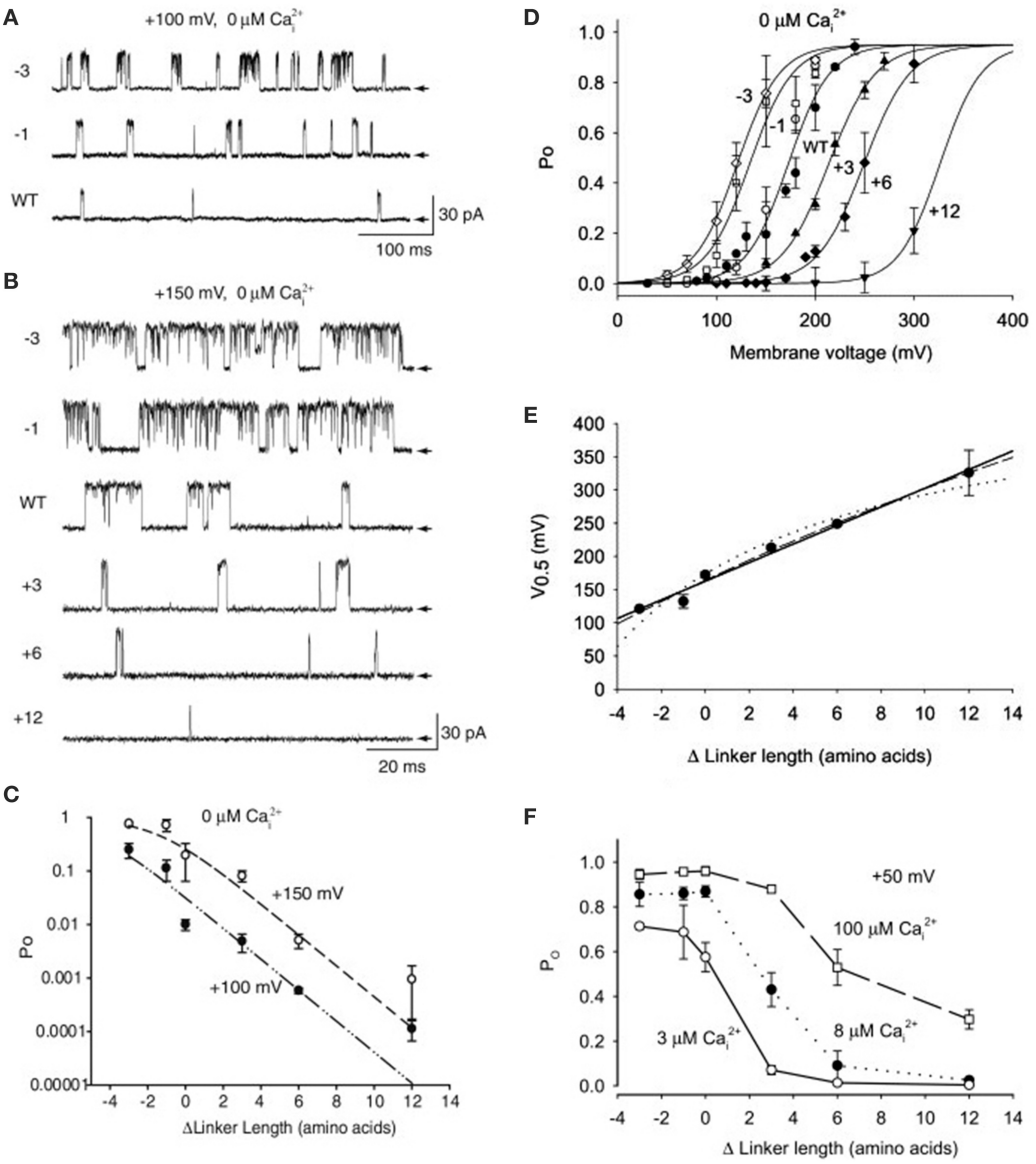
**The linker-gating ring complex applies passive spring force to open the channel in 0 Ca^2+^ and active machine force to open the channel in the presence of Ca^2+^**. **(A,B)** In the absence of Ca^2+^ shortening the RCK1-S6 linkers connecting the gating ring to the PGD (diagrammed in Figure 2) by 1 or 3 amino acids (−1, −3) increased channel activity. Two examples are presented, at +100 and +150 mV. Lengthening the linkers by +3, +6, or +12 amino acids progressively decreased channel activity. The arrows indicate the closed level of the single channel currents. **(C)** Plot of Po vs. change in linker length at two different potentials for data like that in parts **A** and **B. (D)** Shortening the RCK1-S6 linkers shifted the Po vs. voltage plots to the left and lengthening the linkers shifted the plots to the right. The shift in the voltage for half activation gives a quantitative measure of the change in force associated with the change in linker length. **(E)** The plot of the voltage for half activation vs. linker length at 0 Ca^2+^ is approximated by a straight line, indicating that the linker gating ring complex acts as a passive spring. **(F)** Increasing linker length decreased the action of Ca^2+^, with the greatest decrease at low Ca^2+^, suggesting that the linker transmits opening force from the gating ring to the PGD, and that increasing Ca^2+^ increased the force and/or distance of the Ca^2+^-induced movement of the RCK1-S6 linker. Data from Niu et al. (2004).

To explore if the gating ring acts by pulling on S6 in the pore gate domain through the RCK1-S6 linkers, Niu et al. (2004) shortened and lengthened the linkers. In the absence of Ca^2+^, shortening the linkers by -1 or -3 amino acids increased channel activity (Figures 14A–C), shifting the Po vs. voltage curves to the left (Figure 14D), and lengthening the linkers by +3, +6, or +12 amino acids decreased channel activity (Figures 14A–C), shifting the Po vs. voltage curves to the right (Figure 14D). A possible explanation for these observations is that lengthening the linkers decreased Po by decreasing the pull of the linkers on the PGD. The Po could then be restored by increasing the depolarization which increased the pull the voltage sensors applied to the PGD through the S4–S5 linkers (Figure 2). Conversely, shortening the RCK1-S6 linkers would increase the pull on the PGD, which could then be compensated for by applying less depolarization.

In the absence of Ca^2+^, Niu et al. (2004) observed a near linear relationship between V_0.5_ and changes in linker length (Figure 14E). This relationship could be modeled by assuming that the linker-gating ring complex acts as a passive spring in the absence of Ca^2+^, passively pulling on the PGD to bias the channel toward opening in the absence of Ca^2+^. If a passive opening force by the gating ring were not the case then lengthening the linkers should have had little effect on Po, in contrast to the marked decrease in activity that was observed. Further support that the linker-gating ring complex applies a passive opening force to the channel comes from the observations of Budelli et al. (2013) that BK channels without a gating ring have a right shifted Po vs. voltage curve, indicating that more voltage is required to open the channel in the absence of the gating ring, presumably to replace the passive opening force that was applied by the gating ring before it was removed. Budelli et al. also found that removing the gating ring in the absence of Ca^2+^ decreased mean open interval duration, consistent with the proposal that the gating ring applies a passive opening force to the gates in the absence of Ca^2+^. A major structural contributor to the passive spring may be the AC region of the RCK1 domain (Krishnamoorthy et al., 2005).

In the presence of Ca^2+^, changing the RCK1-S6 linker length had a rather different effect than in the absence of Ca^2+^. Niu et al. (2004) observed that lengthening the linkers in the presence of Ca^2+^ decreased the Ca^2+^ dependent increase in Po over a range of Ca^2+^ (Figure 14F). These observations suggest that the linkers are involved in transmitting the action of Ca^2+^ to the PGD. Their further observation that in higher Ca^2+^ that lengthening the linkers gave the least decrease in Po (Figure 14F) and the greatest decrease at low Ca^2+^ suggests that high Ca^2+^ may provide a greater range of movement in the gating ring which could compensate to partially overcome the extra length of the RCK1-S6 linkers. From macro-current analysis Pico (2003) has made some similar observations for the effects of changes in linker length on Ca^2+^ activation.

The observations in the previous sections are consistent with the RCK1-S6 linkers transmitting force from the gating ring to the PGD to activate the channel. In the absence of Ca^2+^ the gating ring applies a passive opening force to the PGD, and in the presence of Ca^2+^ the gating ring applies active opening force. Yuan et al. (2012) have constructed an interpolative movie using a BK gating ring with MthK and K_V_ transmembrane domains to show how Ca^2+^-induced movement in the gating ring may be transmitted to the PGD through the RCK1-S6 linkers.

## Summary of gating mechanism

This brief review of single-channel gating kinetics has presented a few selected single-channel experiments as an introduction to gating mechanism for BK channels. The Ca^2+^-dependent gating and the voltage dependent gating can each be approximated by two-tiered 10-state models with five closed states on the upper tier and five open states on the lower tier (Schemes 4 and 5). The joint activation by Ca^2+^ and voltage is obtained with five repeats of the 10-state voltage activation mechanism, where each repeat has 0, 1, 2, 3, or 4 bound Ca^2+^. Alternatively, the joint activation can be obtained with five repeats of the 10-state Ca^2+^ activation mechanism, where each repeat has 0, 1, 2, 3, or 4 activated voltage sensors. Both of these approaches give the same 50-state two-tiered model comprised of 25 closed states on an upper tier and 25 open states on a lower tier. Adding a tier of flicker closed states to extend the 10-state models to three tiers and 15 states and the 50-state model to three tiers and 75 states improves the description of all the models. Ca^2+^ and depolarization activate BK channels by predominantly increasing the rate of channel opening, with a much smaller decrease in the rate of channel closing. Hence, Ca^2+^ and depolarization activate BK channels by mainly destabilizing the closed states.

To a first approximation, voltage and Ca^2+^ can act relatively independently to activate the channel, consistent with separate modules for voltage activation (VSDs) and Ca^2+^ activation (gating ring), with both allosterically coupling at the PGD (Figure 2). Nevertheless, detailed analysis suggests that there may be interactions within and among the various sensors (Horrigan and Aldrich, 2002; Qian et al., 2006; Shelley et al., 2010). Such interaction might be anticipated because the voltage sensors would be coupled to each other through their attachment to the PGD, so that movement in one VSD might influence movement in the other VSDs. Similarly the Ca^2+^ sensor domains, RCK1 and RCK2, in the gating ring could interact through the gating ring itself and also through their attachment to the PGD. Because VSDs and the Ca^2+^ sensors are both attached to the PGD, then they could interact allosterically through the PGD. Such interaction would be expected for allosteric models of activation and has been observed (Horrigan and Aldrich, 2002).

This review has presented only a brief introduction to gating of BK channels with observations and references restricted in most part to selected single-channel recording and analysis papers. For those who have made it this far and would like to delve deeper into the details of gating mechanism, some of the following papers could be consulted (Cox et al., 1997; Cui et al., 1997; Rothberg et al., 1997; Rothberg and Magleby, 1998a,b, 1999, 2000; Horrigan and Aldrich, 1999, 2002; Horrigan et al., 1999; Cui and Aldrich, 2000; Shi and Cui, 2001; Zhang et al., 2001; Magleby, 2003; Chen et al., 2012), as well as the reviews listed at the beginning of this review.

## Needed studies

A full 50-state model (75 states with the flicker closed states), rather than the subset of states from the 50-state model (Scheme 9), will be needed with rate constants to fully describe the kinetics underlying gating for the joint activation of BK channels by voltage and Ca^2+^. A preliminary study has shown that a highly constrained 50-state model can approximate the single-channel data (Chen et al., 2012). A viable model would describe both the single-channel kinetics and the kinetics of the macro current data, as the average of the single-channel responses gives the macro currents.

Future studies need to address to what extent the gating parameters determined by single-channel analysis can account for the gating currents and the pseudo mono-exponential relaxations of macro currents associated with activation and deactivation following voltage steps (Cox et al., 1997; Horrigan and Aldrich, 1999; Horrigan et al., 1999). To account for mono-exponential relaxations, it was proposed that Ca^2+^ binding and voltage sensing must be fast compared to channel opening and closing (Horrigan and Aldrich, 1999; Horrigan et al., 1999). A test of this proposal and also of the ability of single-channel analysis to estimate parameters typically obtained from voltage jumps could be obtained by using gating parameters estimated from single-channel analysis to predict macro currents for comparison to the observed macro current data.

BK models need to be expanded to take into account the two high affinity Ca^2+^ binding sites on each subunit rather than the usual assumption of one high affinity site. This extension seems essential toward understanding the gating mechanism, as the RCK1 site and Ca^2+^ bowl site may well-act through different mechanisms. This would increase the number of states to 250 and to 375 with a tier of flicker states, but experimental approaches and analysis techniques are available to study these more complete models. Some view such large state models with incredulity, but gating in structurally realistic models with the expected number of states is the same process as gating in simplified models with a limited number of states. No matter the number of states, the channel occupies only one state at any time, and then enters the next state based on the probabilities of the various transition pathways away from the occupied state. If BK channels threw up their voltage sensors and gave up on gating because of disbelief in the large number of potential states available to enter, then our health would surely suffer. With powerful experimental and computational techniques, and fast desktop computers, certainly we can stretch our sights out at least as far as our BK channels do. A 250-state model is just three 10-state models working simultaneously, each of which can be studied separately to obtain the parameters and then combined. Differences between the experimentally observed gating and the gating predicted by a 250-state model would then indicate potential interactions within and among the various domains of the channel, providing further insight into gating mechanism.

## Author contributions

Both authors contributed to writing, revising, and approval of the manuscript.

### Conflict of interest statement

The authors declare that the research was conducted in the absence of any commercial or financial relationships that could be construed as a potential conflict of interest.
